# Coordinated assembly and release of adhesions builds apical junctional belts during *de novo* polarisation of an epithelial tube

**DOI:** 10.1242/dev.191494

**Published:** 2020-12-23

**Authors:** Andrew C. Symonds, Clare E. Buckley, Charlotte A. Williams, Jonathan D. W. Clarke

**Affiliations:** 1 Centre for Developmental Neurobiology, Institute of Psychiatry, Psychology and Neuroscience, King's College London, Guy's Campus, London SE1 1UL, UK; 2 Department of Physiology, Development and Neuroscience, University of Cambridge, Downing Street, Cambridge CB2 3BY, UK

**Keywords:** Adhesions, Epithelial tube, Morphogenesis, Neural tube, Zebrafish

## Abstract

Using the zebrafish neural tube as a model, we uncover the *in vivo* mechanisms allowing the generation of two opposing apical epithelial surfaces within the centre of an initially unpolarised, solid organ. We show that Mpp5a and Rab11a play a dual role in coordinating the generation of ipsilateral junctional belts whilst simultaneously releasing contralateral adhesions across the centre of the tissue. We show that Mpp5a- and Rab11a-mediated resolution of cell-cell adhesions are both necessary for midline lumen opening and contribute to later maintenance of epithelial organisation. We propose that these roles for both Mpp5a and Rab11a operate through the transmembrane protein Crumbs. In light of a recent conflicting publication, we also clarify that the junction-remodelling role of Mpp5a is not specific to dividing cells.

## Abstract (precis)

**Summary:** Using the zebrafish neural tube as a model, we uncover the *in vivo* mechanisms that allow the generation of two opposing apical epithelial surfaces within the centre of an initially unpolarised solid organ.

## INTRODUCTION

Epithelia are one of the fundamental tissue types of the body, forming protective sheets of cells around the outside of the organism and lining the inside surface of many organs or parts of organs. All epithelia are polarised and have an apical and a basal surface that are molecularly and functionally distinct. In some cases, the organisation of the apical surface of an epithelium is generated at the free surface of an embryonic sheet of cells, whereas in others the apical surface emerges from within a dense rod or ball of cells to generate an epithelial tube or cyst (Martin-Belmonte and Mostov, 2008; Datta et al., 2011). Generating an apical surface from within a rod or ball of cells adds an extra layer of complexity to this process, because cells at the centre of these structures have to lose inappropriate connections to generate a free surface. They must also organise that free surface into a typical apical structure comprising a lattice of closely adherent cells connected by apicolateral belts of specialised junctions (typically adherens junctions and tight junctions in vertebrates). A key step, elucidated in epithelial development in flies, is the Crumbs-dependent redeployment of junctional proteins, such as Baz (*Drosophila* Pard3) and E-Cadherin from the apical-most surface of single-sheet epithelial cells to the apicolateral border of the cell (Grawe et al., 1996; Harris and Peifer, 2005; McGill et al., 2009; Morais-de-Sá et al., 2010; Pilot et al., 2006; Tepass, 1996). This builds the apicolateral junctional belts that tie the epithelial cells together (St Johnston and Ahringer, 2010; Wei et al., 2005). The extent to which similar mechanisms of apical surface development are used at a free surface in comparison to within a rod or sphere primordium, and how these can be coordinated with the remodelling of cell-cell connections necessary for *de novo* surface generation, is poorly understood, especially in vertebrates *in vivo*.

Here, we analyse the spatial deployment of the cardinal polarity protein Partitioning-defective 3 (Pard3) and the cell adhesion protein Cadherin 2, type 1, N-cadherin (neuronal) (Cdh2) during generation of the apical surface of the neural tube in the zebrafish embryo *in vivo*. Here, the apical surface is generated *de novo* from within the solid neural rod. The relative accessibility and transparency of zebrafish embryos provides an advantageous system in which to address the mechanism in whole embryos. We use experimental manipulations of the Mpp5a (also known as Nok or Pals1) scaffold protein and the endocytic recycling protein Rab11a. We determine the cellular and molecular mechanisms that release cell adhesions across the organ midline whilst simultaneously generating the canonical apical junctional belt organisation of epithelia within a solid primordium. We compare these with previously shown mechanisms that generate apical organisation at a free surface. We show that both Mpp5a and Rab11a are required to remodel connections between contralateral and ipsilateral cells and suggest that they operate through apical recruitment of the transmembrane protein Crumbs.

## RESULTS

### Apical rings of Pard3 and ZO-1 are built up from the ventral floor plate

**Fig. 1.**
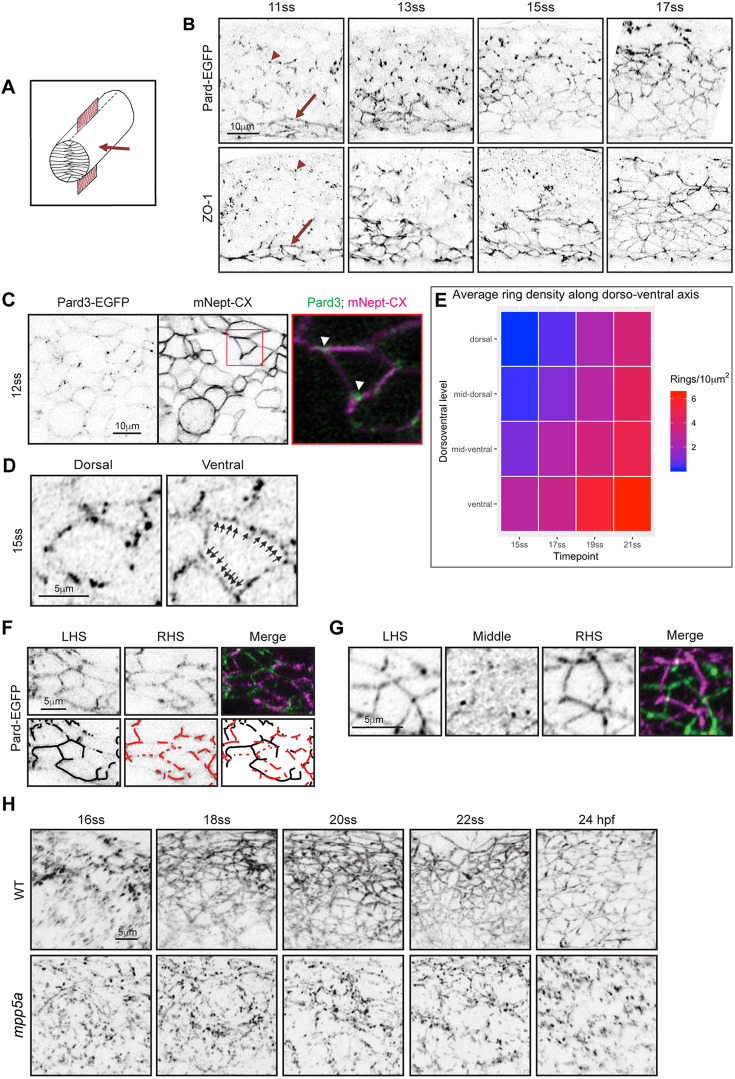
**Mpp5a-dependent transition from spot adhesion to apical ring.** (A) Diagram of neural rod with inserted red sheet to illustrate sagittal plane of confocal sections. Red arrow indicates direction of imaging. (B) Sagittal confocal planes of neural rod in anterior spinal cord region at 11-, 13-, 15- and 17-somite stages (ss). Comparable images were seen at each time point from three embryos. Images in the bottom row are from fixed embryos stained for ZO-1. Comparable images were seen from two embryos at each time point. Red arrow indicates ventral apical rings and red arrowhead indicates puncta. Dorsal is at the top of each panel. (C) Sagittal confocal images at 12-somite stage showing that Pard3-EGFP puncta are often located at cell vertices (arrowheads, *n*=8 embryos). Plasma membrane was imaged using mNeptune2.5-CAAX (mNept-CX). (D) Examples of punctate Pard3-EGFP in developing apical rings from a 15-somite-stage embryo. Dorsal example shows an immature, incomplete ring. Ventral example shows a more complete ring, with arrows indicating multiple puncta between vertices. Single sagittal confocal planes. (E) A heat-map quantification of the formation of mature Pard3 apical rings from two embryos over developmental time and dorsoventral position. (F) Parasagittal confocal sections from left-hand side (LHS) and right-hand side (RHS) of 15-somite stage neural rod. Incomplete apical rings of Pard3-EGFP are forming independently on left and right sides of the midline. (G) Parasagittal and sagittal confocal sections at 17-somite stage showing complete apical rings of Pard3-EGFP on either side of the midline. The sagittal section (labelled Middle) shows a largely diffuse, low level of Pard3-EGFP expression in the midline territory between the left and right rings (see Movie 1). Apical ring formation in F,G was analysed from >10 embryos. For each embryo, 10-25 apical rings were located on one side of the neuroepithelium, and a *z*-stack was taken through the middle of the neural rod until the rings on the opposite side were visible. (H) Images taken from confocal time-lapse movies of wild-type (WT) and *mpp5a* morphant Pard3-EGFP embryos in sagittal orientation from the 16-somite stage to 24 hpf. Comparable images were obtained from three embryos from each genotype. Three control morphants were also assessed in the Cdh2-tFT transgenic line, and all had apical rings comparable to wild types.

The apical surface of epithelia is characterised by a lattice-like arrangement of polarity and scaffolding proteins [such as Pard3, atypical protein kinase C (aPKC) and zona occludens 1 (ZO-1; also known as Tjp1a)] and cell adhesion proteins (such as Cdh2). We visualised this organisation before lumen opening in the sagittal plane of the zebrafish neural rod [stages 10-18 somites, 14-18 h postfertilisation (hpf)] at the level of the nascent anterior spinal cord (Fig. 1A,B). We generated a bacterial artificial chromosome (BAC) transgenic fish line that reports endogenous spatiotemporal expression of Pard3, a cardinal polarity protein, in live embryos [TgBAC(pard3:Pard3-EGFP)^kg301^, referred to as ‘Pard3-EGFP’]. At early rod stages (11 somites), apical rings of Pard3-EGFP and ZO-1 were first established at the midline in ventral floor plate cells (Fig. 1B). In more dorsal areas of neuroepithelium, where apical rings had not yet formed, Pard3-EGFP and ZO-1 were seen as puncta (Fig. 1B, e.g. arrowheads), reminiscent of spot adherens junctions in other systems (e.g. Tepass, 1996). Over the next few hours of rod stage (≤17-somite stage) the lattice work of apical rings progressively builds from ventral to dorsal such that at any time point there is a developmental gradient along the dorsoventral axis (Fig. 1B,E). In contrast to a previous study (Guo et al., 2018), our results suggest that Pard3 is an early component of the nascent apical junction organisation, with both Pard3 and ZO-1 being expressed in a punctate manner during a dynamic phase of cell interdigitation across the neural midline (present results and Buckley et al., 2013).

### Pard3 puncta initially appear at cell vertices

To understand where in the cell Pard3 puncta first appear, we labelled Pard3-EGFP embryos with membrane-tagged mNeptune2.5 and obtained images before apical surface formation. Pard3-EGFP puncta were seen close to the midline around the perimeter of cells, often at cell vertices (Fig. 1C). Membrane-tagged mNeptune was not enriched at vertices, indicating that Pard3-EGFP enrichment is not simply attributable to more membrane at these points. Later, Pard3-EGFP puncta were localised progressively closer together and not confined to cell vertices (Fig. 1D). This suggests that apical rings might be built by adding Pard3 puncta at cell-cell interfaces until they coalesce to form a more-or-less continuous ring structure.

### *mpp5a^m227^* mutants fail to undergo the transition from puncta to apical ring

To uncover mechanisms that regulate formation of apical junctions in the neural rod, we examined puncta-to-apical ring transition in a mutant fish line that fails to open a lumen. Mpp5a is a MAGUK (membrane associated guanylate kinase) scaffolding protein (Wei and Malicki, 2002), essential to retain all forms of the transmembrane protein Crumbs at the apical surface (Zou et al., 2013). *mpp5a* mutants fail to generate and open a lumen in the neural rod and have disorganised adherens junctions (Lowery et al., 2009; Lowery and Sive, 2005). Although *mpp5a^m227^* mutants generated Pard3-EGFP puncta in the sagittal plane at the 16-somite stage, these puncta failed to undergo the puncta-to-apical ring transition and remained as punctate deposits (Fig. 1H). Mpp5a is thus crucial to build apical junctional rings.

### Apical rings on either side of the tissue midline develop independently and are offset from one another

To undergo *de novo* lumen formation successfully, the zebrafish must generate not one, but two lattice-like apical surfaces in the middle of a solid tissue, one on the left side and one on the right side of the future lumen. One possibility is that a single plane of apical rings might first develop at the left-right interface and then these pioneer rings split into left and right rings. To assess this, we analysed development of left and right rings. Imaging sagittal and parasagittal planes, we never saw an embryo containing a single plane of apical rings (Fig. 1F,G; Movie 1). Instead, rings were always found bilaterally, and incomplete apical rings were also found bilaterally on either side of the midline, showing that left and right rings are built independently. Furthermore, the developing rings on either side of the midline are always offset from one another, rather than having a mirror-symmetric arrangement that might be expected if individual rings split into two (Fig. 1G).

### Non-sister connections impose cell offsets across the midline

**Fig. 2.**
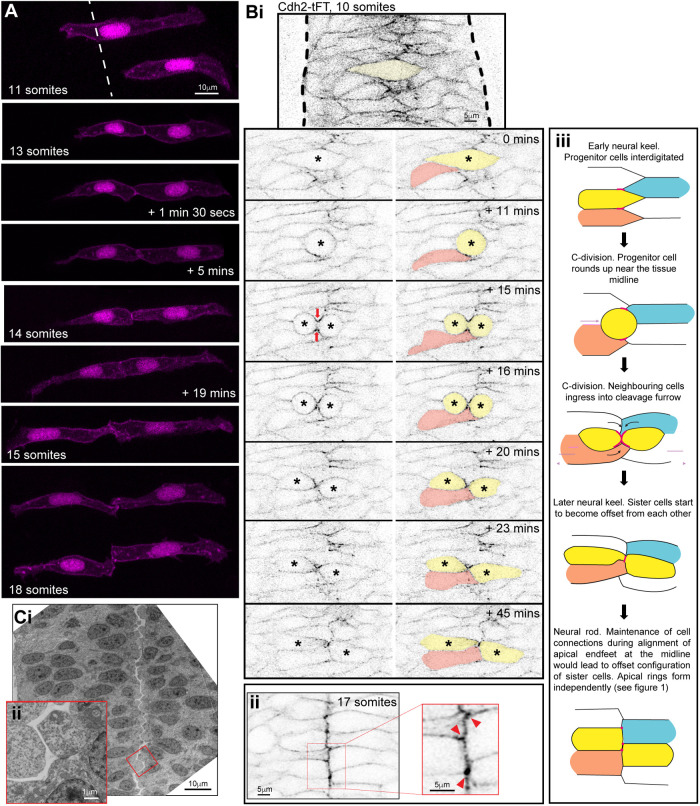
**Sister cells remain attached via their corners.** (A) Images from time-lapse movie projection in dorsal orientation of mosaically labelled neuroepithelial cells in the hindbrain of an 11-somite-stage wild-type embryo. Membrane and nuclei are labelled in magenta. By the 13-somite stage, both cells had undergone C-division, resulting in pairs of sister cells attached across the tissue midline. The top cell pair was followed over time until the 18-somite neural rod stage, when both cell pairs were imaged. The configuration of cell pair connections was assessed from several different experiments at the neural rod stage (∼16-18 somites), and 26 of 31 pairs of cells from five embryos at neural rod stages were found clearly to be attached via their corners. The remaining five were either connected via a more ‘*en face*’ configuration or their configuration was uncertain (e.g. owing to a very thin connection point). (Bi) Single *z*-planes from a time-lapse movie of a C-division (yellow cell) starting at the 10-somite stage (neural keel), from a Cdh2-tFT transgenic embryo. The image contrast was increased in the reference image at the top to highlight that Cdh2 was concentrated at the interdigitation zone between cells around the tissue midline at 0 min. Cdh2-GFP becomes strongly concentrated in the cleavage furrow (time point 15 min), and neighbouring cells ingress into the cleavage furrow (21 of 21 divisions, red arrows). In this example, the pink cell that ingresses into the cleavage furrow gains a contralateral contact with the contralateral daughter of the C-division. As a result of this contact, the contralateral daughter (yellow cell on right) becomes attached to two contralateral cells; one is its sister cell from the C-division and the other is one of its sister's neighbouring cells (the pink cell in this example). (Bii) 5 µm projection of 17-somite stage neural rod from a Cdh2-tFT transgenic embryo. Endfeet are aligned along a centrally located midline. Cdh2-GFP is upregulated along the midline, particularly at cell corners (red arrowheads in magnified region). (Biii) Model depicting the co-ingression of neighbouring cells into the cleavage furrow during C-division (yellow cells) and the subsequent offsetting of sister cells from each other, which precedes apical ring formation, based on images similar to those in Bi. Red lines and dots represent high levels of Cdh2 associated with the dividing cell. The ingression of either ipsilateral (e.g. orange) or contralateral (e.g. blue) neighbours into the cleavage furrow promotes the formation of multiple contralateral connections across the midline. For example, the left-hand yellow sister cell becomes attached to both its right-hand yellow sister cell and the ingressing blue cell. The right-hand yellow sister cell becomes attached to both its left-hand yellow sister cell and the ingressing orange cell. (Ci) Transmission electron micrographs of a 20 hpf embryo hindbrain in transverse orientation. The lumen has started to open from the midline. The interface between contralateral cells has a striking ‘zig-zag’ pattern (three of three 19-20 hpf embryos). (Cii) Inset magnified region from Ci.

Most neural rod cells are derived from progenitor divisions that generate mirror-symmetrically polarised sisters connected to each other across the midline (the C-division; Buckley et al., 2013; Kimmel et al., 1994; Tawk et al., 2007). This process will, at least temporarily, align pairs of sister cells across the midline. However, the offset organisation of left and right apical rings later in development suggests that a mechanism exists to add offset to this initial alignment. To confirm that sister cells become offset, we generated mosaically labelled embryos and used live imaging to assess the interface between sister cells. Soon after a C-division, sister cells appeared mirror-symmetric to each other across the tissue midline (Fig. 2A, 13 somites). However, the shape of nascent apical endfeet changed over time, probably in response to movements and mitoses of neighbouring (unlabelled) cells (Fig. 2A; Movies 2-4). The location of the sister-cell interface in relationship to the rest of the cell shape therefore also changed until the neural rod stage, when the connection between the sisters was stably offset such that nascent apical endfeet of sister cells met at their corners (Fig. 2A, 18 somites). Thus, an initially mirror-symmetric sister-cell alignment is shifted to an offset organisation after C-division mitosis. The offset conformation suggests that contralateral cell connections across the midline are likely not to be confined to their C-division sisters.

To examine the dynamics of ipsilateral and contralateral cell adhesions during the process of the midline C-division, we used a BAC transgenic line expressing the cell adhesion protein Cdh2 fused to a tandem fluorescent timer (Revenu et al., 2014). Cdh2-GFP is expressed in neural rod cells over the entire plasma membrane. Before C-divisions, cells intercalated across the midline, and Cdh2-GFP was enriched in this interdigitation zone, showing that cells contact and adhere to several contralateral, in addition to ipsilateral, cells (Fig. 2Bi). When cells entered C-division mitosis near the midline, cell processes from neighbouring cells ingressed into the cleavage furrow, and distinct enrichments of Cdh2-GFP became largely localised to the cleavage furrow during telophase (red arrows in Fig. 2Bi). This cell ingression allowed neighbouring cells to contact and, presumably, adhere to both ipsilateral and contralateral daughters of the C-division. Cell ingression into the cleavage furrow therefore provides a potential mechanism to promote connections between multiple contralateral and ipsilateral cells (as shown schematically in Fig. 2Biii).

By the 17-somite stage, Cdh2-GFP was enriched at the interface between left and right cells, sometimes at the apical cell vertices (Fig. 2Bii). By this stage, the left-right interface was largely a straight line at the midline. To see whether the offset cell conformation between cells on the left and right persists until lumen opening, we examined electron micrographs taken at the start of lumen inflation. These showed a striking zig-zag conformation between cells separating across the midline (Fig. 2C). It appears that the offset conformation between left and right cells persists until the lumen inflates, separating the left and right sides of the neural rod.

In summary, these data suggest that the C-division can promote connections between multiple ipsilateral and contralateral cells during epithelialisation. We suggest that the maintenance of these connections until rod stage would promote an offset conformation between sister cells.

### A single midline interface enriched in polarity and cell adhesion proteins precedes bilateral apical rings

**Fig. 3.**
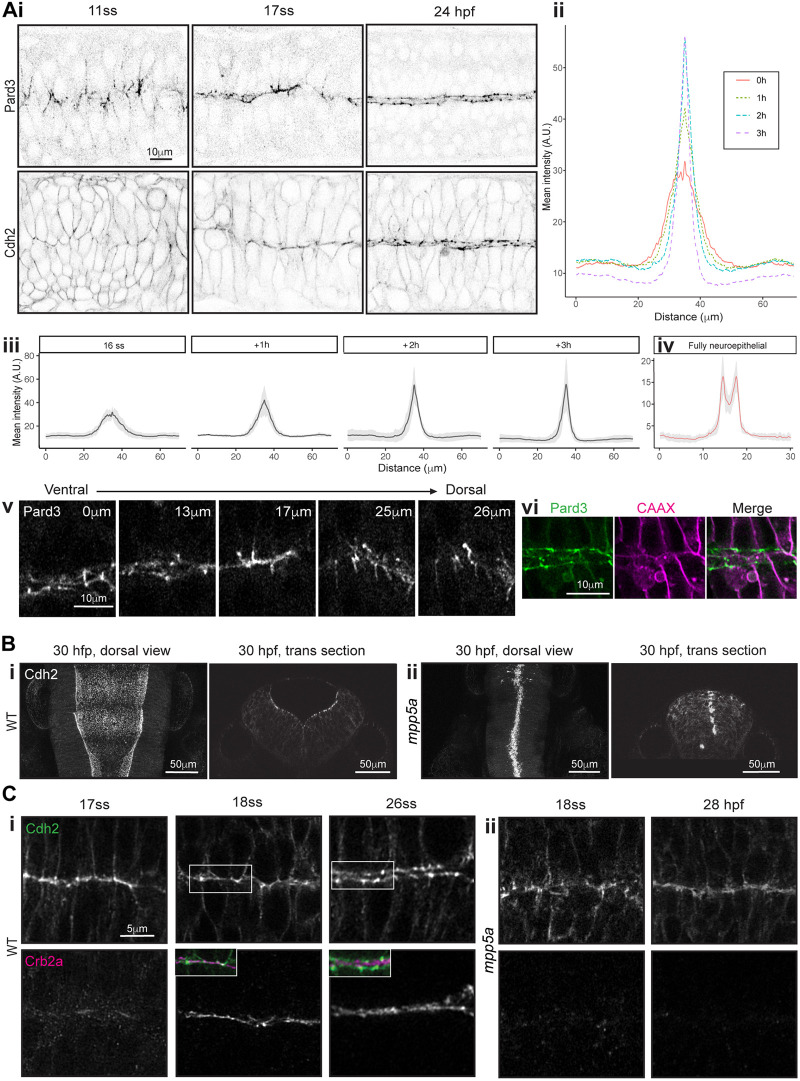
**Mpp5a-dependent remodelling of midline adhesions.** (Ai) Images from confocal time-lapse movies of Pard3-EGFP and Cdh2-GFP embryos in horizontal orientation at 11-somite stage (ss), 17ss and 24 hpf stages. Comparable images were seen from three embryos from each transgenic line. (Aii,Aiii) Mean intensity profiles from six embryos, quantifying Pard3 intensity across the basal-to-basal width of the developing neuroepithelium over time, starting at the 16-somite stage. Standard deviation is shown as a grey ribbon around the line profile for each time point in Aiii. (Aiv) Mean intensity profiles from the same six embryos, quantifying Pard3 intensity across the basal-to-basal width of the neuroepithelium at the fully neuroepithelial stage. (Av) Horizontal confocal planes of 17-somite stage neural rod showing Pard3-EGFP expression at five different dorsoventral levels. The single elevated plane of expression at the left-right interface, seen at the level of 17 μm, lies dorsal to levels where apical rings are already formed and ventral to levels where expression is more prominent in mediolateral streaks. (Avi) Single horizontal plane confocal section of Pard3-EGFP and mNeptune2.5-CAAX and merge at ∼24 hpf. Plasma membranes meet at the tissue midline, and Pard3 is now largely located in two parallel parasagittal domains. (B) Horizontal and transverse confocal sections of 30 hpf. Wild-type (WT; Bi) and *mpp5a^m227^* mutant (Bii) Cdh2-GFP embryos at the hindbrain level. The hindbrain lumen remained closed in five of five *mpp5a^m227^* mutant embryos and is always open in wild types. (C) Horizontal confocal sections of wild-type (Ci) and *mpp5a^m227^* mutant (Cii) Cdh2-GFP embryos at the anterior spinal cord level, stained for Crb2a. Insets in the 18- and 26-somite stages of wild types show merged images of Crb2a and Cdh2-GFP expression. (Ci) In wild-type embryos, Cdh2 and Crb2a were colocalised at the midline at the 18-somite stage (four of four embryos), but Cdh2-GFP was displaced basolaterally to form two independent stripes of expression either side of the midline by the 26-somite stage (eight of eight embryos). In *mpp5a^m227^* mutants, Crb2a was not present at the midline at the 18-somite stage (four of four embryos), and Cdh2-GFP remained in a single expression domain at the tissue midline even as late as 28 hpf (five of five embryos).

To understand the transition from spot-like junctional specialisations to two independent lattices of apical rings on left and right sides of the midline, we analysed the distribution of Pard3 and Cdh2 during this transition. Given that there is a developmental gradient along the dorsoventral axis of the neuroepithelium (Fig. 1B,E), we focused on a mid-dorsoventral plane of the anterior spinal cord. We imaged Pard3-EGFP and Cdh2-GFP from neural keel to neural rod stages in the horizontal plane (Fig. 3Ai-Aiii). Pard3-EGFP and Cdh2-GFP initially had different patterns of expression. At 11 somites (14 hpf), puncta of Pard3-EGFP were localised broadly around the midline, along interfaces between interdigitating cells. These correspond to the spot distribution of Pard3-EGFP seen sagittally (Fig. 1B,C). At the same time point, Cdh2-GFP was expressed at low levels throughout the cell membrane but also slightly elevated in the interdigitation zone, mostly in discrete spots. By 17 somites (17.5 hpf), both Cdh2-GFP and Pard3-EGFP were clearly enriched at the neural rod midline in a near-continuous, fairly straight expression domain at the interface between left and right cells. Consistent with a developmental gradient along the dorsoventral axis of the neuroepithelium, dorsal to this plane, Pard3-EGFP was still expressed in a discontinuous mediolateral stripe pattern, and ventral to this it appeared in two parasagittal domains either side of the midline (Fig. 3Av). The intermediate level that shows a single left-right interface of Pard3-EGFP expression occupies a small and transient dorsoventral domain that moves dorsally as the assembly of bilateral rings progresses from ventral to dorsal.

To analyse the distribution of cell membranes relative to the expression of Pard3-EGFP, we revealed membranes by injecting embryos with mRNA mNeptune2.5-CAAX. By 24 hpf, although spinal cells at almost all dorsoventral levels still met at the left-right interface (see Fig. 3vi), Pard3-EGFP and Cdh2-GFP were no longer localised to this interface; instead, their expression had transitioned from mediolaterally arranged puncta through a single midline domain and into two parasagittal domains either side of the midline (Fig. 3Ai,Aiv). These two domains were characterised by periodically elevated spots of expression that corresponded to points within the left and right apical rings. Much lower levels of Pard3-EGFP and Cdh2-GFP remained in the medial zone between bilateral apical rings (Fig. 3Ai,Aiv).

### Mpp5a is required to redeploy adhesions to the apicolateral border

Although eliminating Crumbs in zebrafish is difficult because there are multiple Crumbs proteins, we took advantage of the *mpp5a^m227^* mutant that is unable to localise Crumbs proteins to the apical surface (Zou et al., 2013). Given that we demonstrated that the apical spot-to-ring transition did not occur in *mpp5a^m227^* mutants (Fig. 1H), we wanted to test the role of Mpp5a in displacing Pard3 and Cdh2 proteins from a single line at the left-right interface to the bilateral expression domains seen at later time points. For this, we generated a *mpp5a^m227^* mutant; Cdh2-tFT (Cdh2-tandem fluorescent protein timer) compound transgenic line. The ‘wild-type’ Cdh2-tFT embryos opened a lumen in the hindbrain and anterior spinal cord (Fig. 3Bi), whereas *mpp5a^m227^*; Cdh2-tFT embryos failed to open a lumen (Fig. 3Bii), in line with previous observations of *mpp5a* mutant embryos (Lowery and Sive, 2005).

To understand the *mpp5a^m227^* phenotype in relationship to Cdh2 remodelling and its relationship to Crumbs expression, we examined their localisation in wild-type and *mpp5a^m227^* embryos. In wild-type embryos (Fig. 3Ci), Crb2a was first expressed at the neural rod 17- to 18-somite stage at the left-right interface, coincident with a single line of expression of Cdh2. However, by 26 somites, when Crb2a was still localised to the left-right interface, Cdh2-GFP protein was displaced to form two slightly more basolateral lines of expression. Cdh2-GFP expression was located immediately lateral to Crb2a, with little overlap of expression.

In *mpp5a^m227^* mutant embryos (Fig. 3Cii), Crb2a was absent from the midline, as expected. At the 18-somite stage, *mpp5a^m227^* mutant Cdh2-tFT embryos showed a single domain of enrichment of Cdh2-GFP at the left-right interface, similar to wild-type Cdh2-GFP embryos. However, unlike wild-type embryos, Cdh2-GFP remained as a single midline domain in *mpp5a^m227^* mutants, failing to undergo the transition to two lines of Cdh2-GFP either side of the midline, even up to stages as late as 28 hpf.

Together, these data show that left and right cells initially adhere together across the midline through contralateral adhesions. However, both Cdh2 and Pard3 are then redeployed more basally to the apicolateral border, where they contribute to adhesive junctions between ipsilateral cells to build coherent, bilateral sheets of neuroepithelium. This redeployment of junctional proteins depends on Mpp5a and is likely to be a crucial step in releasing cells from their contralateral adhesions to allow lumen formation.

### Persistent adhesions might contribute to lack of epithelial maintenance in Mpp5a- and Rab11a-deficient epithelia

**Fig. 4.**
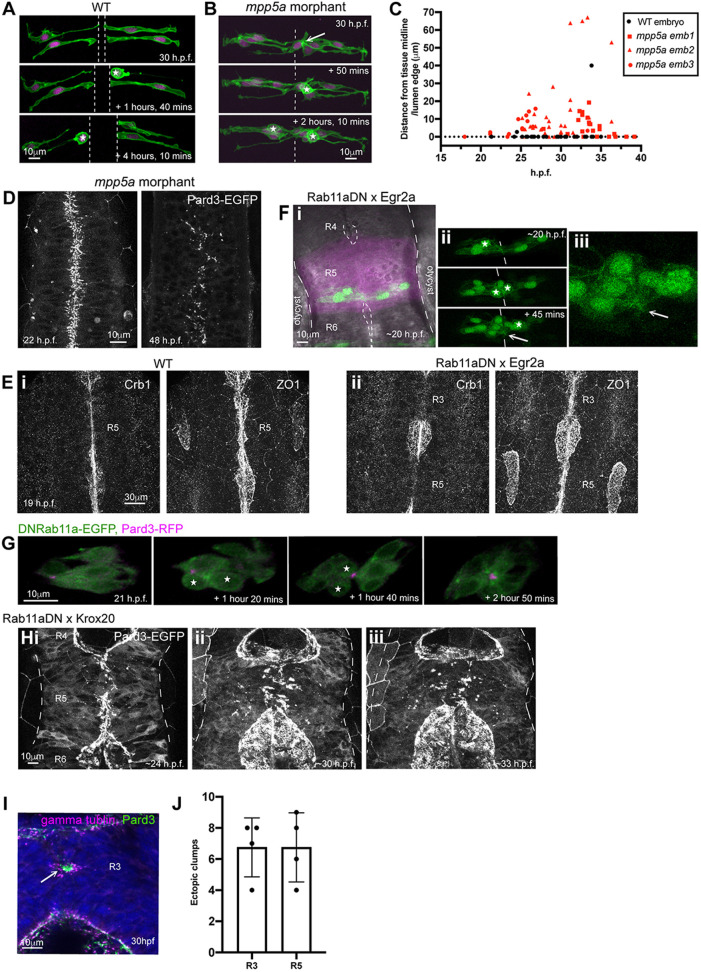
**Persistent adhesion in Mpp5a- and Rab11a-deficient cells.** (A) Images from time-lapse movie of mosaically labelled neuroepithelial cells in the hindbrain of a 30 hpf wild-type (WT) embryo, dorsal view. Cells have separated across the tissue midline and the lumen has opened (dashed lines). D-divisions (stars) occur at the apical surface, and cells re-established an elongated morphology towards the basal surface after division. (B) Images from time-lapse movie of mosaically labelled neuroepithelial cells in the hindbrain of a 30 hpf *mpp5a* morphant embryo, dorsal view. Cells have failed to separate across the tissue midline (dashed line) and have formed clumps (sometimes with a rosette-like structure; arrow). D-divisions (stars) occurred near the centre of the cell clumps, which was often not situated near the tissue midline. Of 117 daughter cells followed post-division, ≥40% did not re-extend fully to the basal side of the neural rod. (C) Graph of cell division locations in relationship to the tissue midline or lumen edge over development. Seventy-one cells were analysed from three *mpp5a* morphant embryos. In embryos >25 hpf, 49% of *mpp5a* morphant cells divided 5 µm or more away from the midline. Division locations from a single wild-type embryo example are included in black. (D) A 10 µm *z*-projection, dorsal view, at mid-dorsoventral level through the hindbrain of *mpp5a* morphant Pard3-EGFP embryos at 22 and 48 hpf. At 22 hpf, Pard3-EGFP was localised near the tissue midline but did not form continuous straight expression domains as seen in wild types (Fig. 3Ai), and the lumen failed to open. By 48 hpf, Pard3-EGFP localisation became fragmented into clumps. Nine of nine *mpp5a* mutant embryos and six of eight *mpp5a* morphant embryos >24 hpf old from five different experiments had fragmented midlines and ectopic apical proteins. The extent of this disorganisation was greater in older embryos. Two of six *mpp5a* morphant embryos had a milder phenotype (see Fig. S1). Nine of nine wild-type embryos had normal apical surfaces, with no ectopic apical proteins. (E) 70-80 µm maximum projections, dorsal view of hindbrain at 19 hpf, stained for Crb1 and ZO-1. (Ei) Wild-type embryo. Both Crb1 and ZO-1 were localised to the apical midline (*n*=3/3). (Eii) Embryo in which UAS:DNRab11a was expressed under the Egr2a:KalTA4 activator in rhombomeres (R) 3 and 5. ZO-1 localised to the midline, but Crb1 was largely absent from rhombomeres 3 and 5 (*n*=3/3). (Fi) 11 µm maximum-intensity projection of dorsal view of hindbrain neuroepithelial cells at 20 hpf in a DNRab11a × Egr2a embryo. Magenta shows DNRab11a rhombomere 5. Lumen in rhombomere 5 failed to open but had opened in rhombomeres 4 and 6 (short-dashed lines are lumen surface, long-dashed lines are basal surface). (Fii) Maximum-intensity projection images from time-lapse movie of cells in Fi, which failed to separate across the tissue midline (white dashes). Similar to *mpp5a* morphant embryos (B), after D-divisions (stars), cells remained attached and were arranged into a rosette-like structure (arrow), enlarged in Fiii. Cell clumping or rosette-like structures were observed in DNRab11a rhombomeres of all nine embryos analysed from three experiments. Note, in all panels in F we have edited out overlying cells from *z*-planes that would otherwise obscure the view of the cells participating in the rosette. Unedited versions of these images can be seen in Fig. S1. (G) Time-lapse reconstruction showing mosaically distributed DNRab11a-EGFP neuroepithelial cells in hindbrain of 24-somite-stage (21 hpf) embryo. After D-divisions (stars), cells did not separate and formed a rosette around centrally located puncta of Pard3-RFP. (H) 70 µm maximum projections from time-lapse movie through a DNRab11a × Egr2a embryo hindbrain labelled with Pard3-EGFP, starting at 24 hpf. As in *mpp5a* morphants in D, Pard3 was initially close to the midline of rhombomere 5, but the lumen failed to open and Pard3 localisation became fragmented into clumps that dispersed over time (17 of 17 embryos). (I) 4 µm maximum projection, dorsal view of 30 hpf DNRab11a × Egr2a embryo rhombomere 3, stained for Pard3 and gamma-tubulin. Pard3 is localised in a round clump, surrounded on all sides by centrosomes. (J) DNRab11a rhombomeres 3 and 5 had approximately seven ectopic clumps of Pard3 and gamma-tubulin at 30 hpf (*n*=4 embryos). Error bars denote standard deviations. No ectopic clumps were seen in wild-type rhombomeres.

To test whether Mpp5a-deficient cells retain persistent adhesions across the midline, we analysed the behaviour of clonally related cells in the hindbrain regions of 25 hpf wild-type embryos that had open lumens and *mpp5a* morphant embryos that were unable to open a lumen. In wild-type embryos, cells successfully separated across the midline after C-division; they maintained an elongated morphology that stretched from the apical to the basal surface, and later differentiative divisions (D-divisions) occurred at the apical surface (Fig. 4A). However, in *mpp5a* morphant tissue, cells were not separated across the midline, they often did not extend fully to the basal surface and they appeared clumped together, often in rosette-like structures. D-divisions in *mpp5a* morphants occurred within these cell clumps, often at a distance from the midline of the tissue (Fig. 4B,C). The behaviour of ectopic divisions in *mpp5a* morphants is accompanied by a lateral dispersal of Pard3 from the midline (Fig. 4D). Thus, in addition to being unable to undergo the puncta-to-apical ring transition (Fig. 1H), Mpp5a-deficient cells show persistent adhesions, and apical midline organisation becomes progressively fragmented over time.

Given that we hypothesise that Mpp5a functions through its ability to localise Crumbs to the nascent apical surface, we next analysed cell behaviours after another manipulation that depletes Crumbs from the apical surface. We expressed a dominant-negative (DN) form of Rab11a in rhombomeres 3 and 5. We previously showed that this manipulation reduces apical Crb2a localisation and prevents lumen opening in these segments (Buckley et al., 2013). Here, we show that apical Crb1 is also downregulated in DNRab11a rhombomeres (Fig. 4E), confirming that this manipulation is sufficient to downregulate apical trafficking of multiple Crumbs paralogues. Cells in DNRab11a rhombomeres did not separate after D-division and formed cell clumps and rosette-like structures with a central focus of Pard3 (Fig. 4F,G; Fig. S1). Similar to *mpp5a* morphants (Fig. 4D), a lateral dispersal of Pard3-EGFP from the midline was seen, progressively fragmenting midline organisation over time (Fig. 4H). This supports our previous observations of ZO-1 dispersion in DNRab11a segments (Buckley et al., 2013). Staining of DNRab11a rhombomeres revealed ectopic clumps of Pard3 and centrosomes, sometimes arranged in rosette-like structures, with centrally located Pard3 surrounded by centrosomes (Fig. 4I,J).

**Fig. 5.**
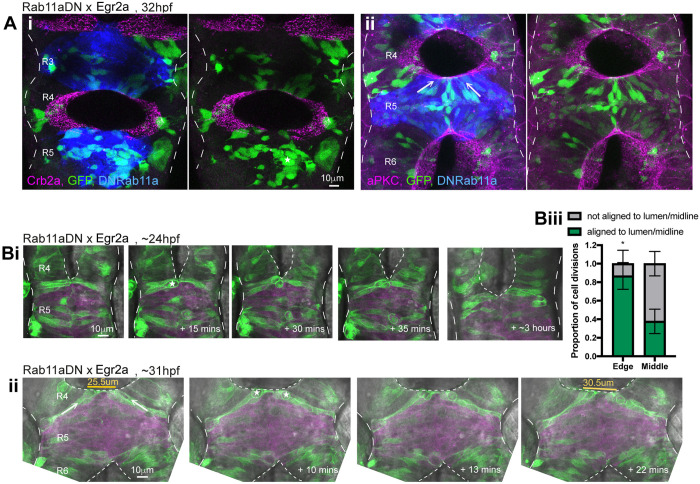
**Cells at the wild-type/DNRab11a interface contribute to the luminal surface.** (A) 15-18 µm maximum projections through 32 hpf DNRab11a × Egr2a embryo hindbrains stained for Crb2a (Ai) or aPKC (Aii). Cells were mosaically labelled with cytoplasmic GFP and H2A-GFP. (Ai) Crb2a is largely absent from DNRab11a cells (blue) contacting the lumen (five of five embryos). (Aii) aPKC is present at the lumen in both wild-type and DNRab11a cells (blue) (four of four embryos). Cells in the centre of DNRab11a rhombomeres (R) clumped together (e.g. star in Ai), whereas cells in contact with the open lumens had elongated morphology (e.g. arrows in Aii). (B) Single dorsal view *z*-slices from time-lapse movie of neuroepithelial cells in rhombomere 5 of a 24 hpf DNRab11a × Egr2a embryo. Long-dashed lines denote basal surfaces. Short-dashed lines denote apical surfaces. (Bi) As the neighbouring lumen inflated, cells at the edge of the DNRab11a rhombomere remained connected across the midline, and cells near the edge divided at this central point of connection with parallel orientation (star). (Bii) As the neighbouring lumen inflated further, cells near the wild-type/DNRab11a interface reoriented towards the open lumen (e.g. arrows). Cells divided at the opening luminal surface with parallel orientation (stars). This widened the DNRab11a luminal surface further (see measurements in orange). (Biii) Quantification of cell division orientation. One hundred and four DNRab11a cell divisions were analysed from three embryos over the 24-40 hpf period of development. Eighty-seven per cent of DNRab11a cells dividing at the luminal edge did so parallel to the opening lumen, whereas 38% of DNRab11a cells dividing in the middle of rhombomeres 3 and 5 did so parallel to the midline (*P*=0.0121, Student's unpaired, two-tailed *t*-test). Error bars denote standard deviations between embryos.

Interestingly, although cells situated in the middle of closed-lumen DNRab11a rhombomeres 3 and 5 formed clumps, cells at the edges of these rhombomeres often had a typical elongated neuroepithelial morphology. These were able to build an aPKC-positive apical surface that was contiguous with the wild-type apical surface in adjacent open-lumen wild-type rhombomeres 2, 4 and 6 (Fig. 5A). Edge cells were oriented obliquely along the anterior-posterior axis of the embryo, apical towards the open lumens (e.g. arrows in Fig. 5Aii). To determine how this morphology arose, we made time-lapse movies of DNRab11a/wild-type interface neuroepithelial cells. We found that interface cells remained connected across the midline as the neighbouring wild-type lumen inflated, acting like a hinge and reorienting cells at the DNRab11a/wild-type rhombomere boundary towards the opening lumen (Fig. 5Bi). Despite persistent connections between cells, the DNRab11a luminal surface expanded via cell division and subsequent reintegration into the epithelium (Fig. 5Bii). Cell divisions in the centre of DNRab11a rhombomeres were disorganised and did not orient along the tissue midline, whereas those occurring at the DNRab11a luminal surface occurred with parallel orientation (Fig. 5Biii). These results demonstrate that DNRab11a cells at the DNRab11a/wild-type interface are able to reintegrate successfully into the epithelium after division if they have access to an apical lumen surface.

Our data suggest that persistent adhesions between cells in Mpp5a-deficient and DNRab11a embryos both inhibit lumen formation and promote the formation of local clumps of cells. This results in the progressive fragmentation of midline organisation over successive rounds of division in Mpp5a-deficient and DNRab11a embryos.

### Mpp5a is necessary for apical ring formation even in the absence of cell division

A recent analysis of cell divisions and lumen formation in *mpp5a*^*m520*^ mutants suggested that Mpp5a function in lumen formation might be related specifically to the resolution of connections between sister cells after the C-division (Guo et al., 2018). When analysing junction formation in *mpp5a*^*m227*^ mutants, we discovered that the loss of lumen formation itself in these mutants was more complicated than expected. In line with previous observations (Lowery et al., 2009; Lowery and Sive, 2005;), we found the loss of lumen formation was not fully penetrant and we consistently found that small lumens formed at the level of the midbrain-hindbrain boundary (Fig. S2A). Less frequently, small lumens also formed in the dorsal aspects of the hindbrain at the level of the otic vesicles (Fig. S2B). We found that Crb2a protein was present at the apical surface of these lumens (Fig. S2A), consistent with the role of Crumbs in the successful formation of an apical surface and opening of a lumen. The ventricular walls of these small lumens were lined with Pard3-EGFP apical rings (Fig. S2B). We suggest the results of Guo et al. (2018) might be a consequence of their use of the weaker *mpp5a*^*m520*^ mutant allele having non-penetrant phenotypes, as they previously published (Zou et al., 2013). We also suggest that the mini lumen at the midbrain-hindbrain boundary that we observed in *mpp5a*^*m227*^ mutants (Fig. S2) is attributable to the expression of a second Mpp5a-like gene expressed in this region (such as *mpp2b*, Thisse et al., 2004). Mpp5a is a member of the Mpp MAGUK protein family, and there are six MPP genes (*mmp2*, *mmp3*, *mmp4*, *mmp5*, *mmp6* and *mmp7*) with conserved protein domain structure in mammals. All these proteins contain a PDZ (PSD-95/Dlg-A/ZO-1) domain that, in the case of Mpp5, has been shown to be the specific domain that binds Crumbs (Roh et al., 2002). Little is known about the other MPP genes in zebrafish and this could form the basis of further study.

**Fig. 6.**
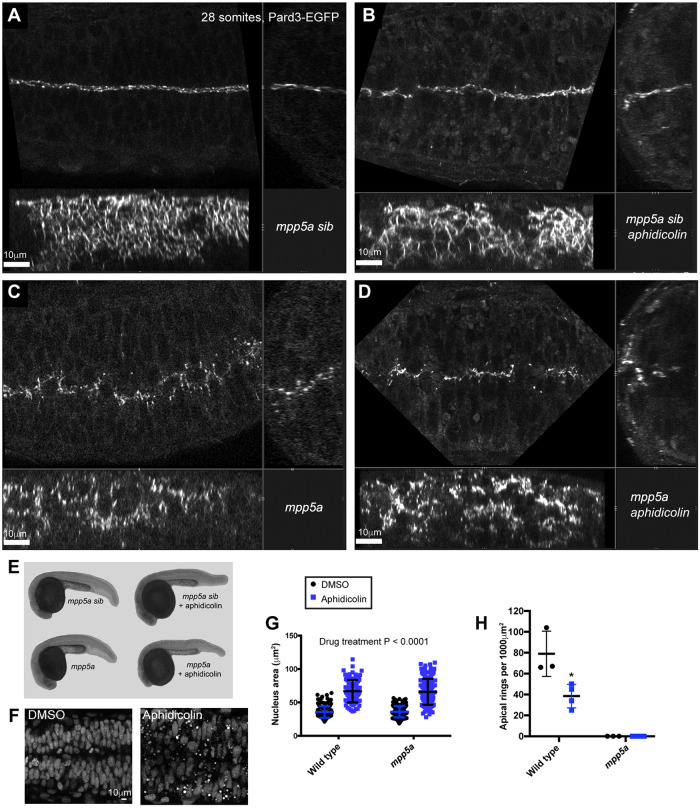
**Mpp5a is necessary for apical ring formation even in the absence of cell division.** All embryos are at the 28-somite stage. (A-D) Orthogonal series of horizontal (top left), transverse (top right) and sagittal (bottom) confocal planes of Pard-EGFP expression in the following embryos. (A) Wild-type sibling treated with DMSO vehicle control. Many small apical rings have formed at the tissue midline (*n*=3/3 embryos). (B) Wild-type sibling treated with aphidicolin to block the C-divisions. Fewer, large apical rings have formed at the tissue midline (*n*=4/4 embryos). (C) *mpp5a^m227^* mutant embryo treated with DMSO vehicle control. No apical rings have formed at the tissue midline. Pard3 is visible as spots along the midline plane (*n*=3/3 embryos). (D) *mpp5a^m227^* mutant embryo treated with aphidicolin to block C-divisions. No apical rings have formed at the tissue midline. Pard3 is visible as spots along the midline plane (*n*=4/4 embryos). (E) Overall body shape of wild-type siblings and *mpp5a^m227^* mutants with and without aphidicolin treatment. (F) Horizontal section through neural tube showing nuclear staining in wild-type embryos treated with DMSO or aphidicolin to block C-divisions. Aphidicolin-treated embryos have larger nuclei, demonstrating that S-phase division block was successful. (G) Quantification of the area of the nucleus of wild-type siblings and *mpp5a^m227^* mutants with and without aphidicolin treatment. Data from 100-150 nuclei were pooled from two or three embryos in each group and analysed by two-way ANOVA. Areas of aphidicolin-treated nuclei were, on average, 30 µm^2^ bigger than those of DMSO-treated embryos (*P*<0.0001). There was no significant difference in the area of nuclei between wild-type and *mpp5a^m227^* mutant embryos (*P*=0.8561). Error bars denote standard deviations. (H) Quantification of the number of apical rings in wild-type siblings and *mpp5a^m227^* mutants with and without aphidicolin treatment. Numbers of apical rings per 1000 µm^2^ were calculated from three or four embryos per group. No apical rings were seen in any of the *mpp5a^m227^* mutants. There were, on average, 40 fewer apical rings per 1000 µm^2^ in aphidicolin-treated wild-type embryos than in DMSO-treated wild-type embryos (Student's unpaired *t*-test, *P*=0.0222). Error bars denote standard deviations.

To address whether Mpp5a function might be related specifically to the resolution of connections between sister cells (as suggested by Guo et al., 2018), we analysed the key events of apical ring formation in *mpp5a*^*m227*^ mutant embryos with and without C-divisions. Given that the closed-lumen phenotype is not fully penetrant in some hindbrain regions of the *mpp5a* mutants (Fig. S2)*,* we suggest that using apical ring formation in the anterior spinal cord, rather than lumen opening in more anterior regions, is a better test of function. We blocked C-divisions using the S-phase inhibitor aphidicolin and analysed Pard3-EGFP in the anterior spinal cord region. Apical rings of Pard3-EGFP were not rescued by blocking C-divisions in *mpp5a*^*m227*^ mutants (Fig. 6). The efficacy of the division block was confirmed by the enlarged nuclear size in aphidicolin-treated embryos (Fig. 6F,G) and by the fewer enlarged apical rings in the aphidicolin-treated siblings (Fig. 6B,H). These results demonstrate that, although necessary for cell-cell separation after division, Mpp5a function is not specifically related to remodelling adhesions between C-division sisters. Instead, it plays a more fundamental role in the formation of apical junctional rings and release of contralateral adhesions during *de novo* polarisation of an epithelial tube.

## DISCUSSION

The development and remodelling of apical epithelial junctions is likely to be regulated by a complex integration of molecular interactions involving polarity proteins and adhesion proteins (Martin-Belmonte and Mostov, 2008; St Johnston and Ahringer, 2010; Datta et al., 2011), together with biomechanical forces that interpret tissue tensions generated by the actomyosin cyctoskeleton (Pinheiro and Bellaiche, 2018). Here, we have investigated some of the cellular and molecular features that underlie *de novo* apical surface generation within the solid rod primordium of the embryonic zebrafish central nervous system. Our work uncovers three aspects of *de novo* apical surface generation within a complex organ *in vivo*. First, despite the very different cellular organisations, apical surface generation at the centre of a solid organ primordium follows a strikingly similar sequence of spot-to-ring junction building to those previously described for apical surface generation at a free surface of a sheet of cells. Second, the displacement of proteins from the nascent apical surface to the nascent apicolateral junctional belt domain serves two purposes in a solid organ primordium; simultaneously, it contributes to the building of epithelial junctional belts and releases adhesions with contralateral cells that would otherwise inhibit the generation of a free surface and formation of a lumen. Third, neuroepithelial cell divisions across the nascent apical plane (C-divisions) do not promote exclusive connections between contralateral sister cells. Instead, connections between multiple ipsilateral and contralateral partners are promoted by neighbour ingression into the cleavage furrow of the C-division and appear to contribute to the offset alignment of contralateral cells across the midline. Through this realignment, sister cells do not remain connected ‘*en face*’, as previously suggested (Guo et al., 2018), but instead the sisters become attached in a configuration offset from one another across the midline. In addition, we address a recent suggestion that the MAGUK scaffolding protein Mpp5a is only required to remodel apical protein location after cell divisions across the nascent apical plane (Guo et al., 2018). We show that this is not the case, because Mpp5a-dependent apical protein remodelling is required for apical surface generation both with and without cell divisions.

### Roles of Mpp5a and Rab11a

We describe a similar displacement of Pard3 and Cdh2 to the apicolateral border of zebrafish neuroepithelial cells to that seen in *Drosophila* ectodermal cells (Grawe et al., 1996; Harris and Peifer, 2005; McGill et al., 2009; Morais-de-Sá et al., 2010; Pilot et al., 2006; Tepass, 1996). In the zebrafish neural rod, the displacement occurs at the midline within a solid rod of cells rather than at a free surface, but despite this fundamental difference in tissue architecture, a similar mechanism appears to build the apicolateral junctional belts. We show that the scaffold protein Mpp5a is necessary for the displacement of Pard3 and Cdh2 and for the formation of apical rings. In line with the *Drosophila* literature mentioned above, we propose that Mpp5a operates through its role in scaffolding Crumbs to the apical surface. This is consistent with the appearance of Crumbs at the nascent apical surface when Pard3 and Cdh2 are displaced from this surface and the lack of Crumbs localisation to the nascent apical surface in *mpp5a* mutants (Fig. 3C; Zou et al., 2013). A direct test of Crumbs function is difficult in zebrafish because three different Crumbs proteins (Crb1, Crb2a and Crb2b) are present at this stage in the developing neural rod. Previous research has shown that neuroepithelial organisation was altered only when all paralogues of Crumbs protein were knocked down by multiple morpholinos (Zou et al., 2013) and, consistent with our hypothesis, this appears to include a lack of lumen formation. Rather than transiently knocking down all paralogues of Crumbs proteins in this study, we used a Mpp5a mutation (*mpp5a^227^*) that downregulates all paralogues of Crumbs protein from the nascent apical surface. In this case, neither Pard3 nor Cdh2 was displaced from the nascent apical surface, and this resulted in persistent adhesions between left and right sides of the neural rod and a loss of lumen formation. Additionally, we found that a stable genetic manipulation of Rab11a that also lacks Crumbs at the nascent apical surface (Roeth et al., 2009; Buckley et al., 2013) also showed persistent adhesions between contralateral cells, a lack of lumen opening and, at later stages, comparable disorganisation of neuroepithelial tissue to *mpp5a* mutants (Fig. 4).

Despite its function in mediating epithelial cohesion via homophilic interactions of its extracellular domain (Thompson et al., 2013), the role of Crumbs protein in allowing cells to resolve their apical adhesions is emerging as another important mechanism for the control of epithelial morphogenesis. For example, a recent study demonstrated that a secreted version of Crb2 acted in a non-cell-autonomous manner to cause delamination of neuroepithelial cells during dorsal collapse of the spinal cord central canal in mouse embryos (Tait et al., 2020). By studying dominant-negative Rab11a cells in an environment where they have access to wild-type neighbours, we uncovered that cells depleted of Crumbs from their apical surface can nonetheless form an epithelial surface when adjacent to wild-type cells. Thus, in the fish neuroepithelium, Rab11a and Crumbs appear to be required for *de novo* epithelial surface generation but dispensable for integration next to an already generated apical surface. This suggests that non-cell-autonomous rescue from neighbouring wild-type cells is able to initiate epithelialisation in the absence of Rab11a/Crumbs. It would be interesting to determine whether secreted Crumbs protein plays a role in this rescue.

### Coordinating left-right release with epithelialisation

The zebrafish neural rod is initially a solid primordium, meaning that the displacement of Pard3 and Cdh2 is not from a free apical surface but instead occurs within the densely packed cell-cell interfaces that lie at the tissue centre. Cell adhesions between contralateral cells across the neural rod initially bind left and right halves of the neural rod together, but these adhesions need to be released to generate the lumen and apical surface of the neuroepithelium. We show that Mpp5a neatly coordinates the release of contralateral connections with the strengthening of ipsilateral connections, thus leading both to the generation of a free surface within the centre of an initially solid tissue and to junctional belt formation and epithelialisation. Mpp5a-deficient cells were unable to form a coherent sheet of apical rings (Fig. 1H). Additionally, in the absence of Mpp5a or the presence of DNRab11a, cells were unable to release cell-cell adhesions. During epithelialisation, these defects first resulted in closed lumens (Figs 3B and 4Hi). This is in line with our previous findings (Buckley et al., 2013) and the findings of others that apical localisation of Rab11 is necessary for cell abscission and normal opening of Kupffer's vesicle in zebrafish (Rathbun et al., 2020). It would be interesting to know whether similar mechanisms also operate in the caudal segments of avian and mammalian spinal cord. The neural tube in these segments forms through the process of secondary neurulation that also involves the *de novo* formation of a central lumen within a condensing rod of cells (see Schoenwolf, 1984, Dady et al., 2014), rather than epithelial folding that generates the neural tube in more rostral segments of these animals.

At later stages of development in the zebrafish neural tube, when neuroepithelial cells undergo interkinetic nuclear migration and D-divisions, the absence of Mpp5a or the presence of DNRab11a resulted in progressive disintegration of midline organisation (Fig. 4D,H). This might be attributable to a combination of factors; during cell division, interkinetic nuclear migration and mitotic rounding pull cells around within the tissue. In a normal epithelium, the apical surface would be stabilised by the lattice of ring-like junctional belts that hold the apical surface together. But when junctional belts between ipsilateral cells are not present, the tissue fails to hold cells at the apical plane, and cells are unable to re-extend fully to the basal side of the neural rod after division. Additionally, a lack of cell separation after division promotes the formation of local clumps of cells. Together, these defects cause divisions to occur in ectopic locations, and the midline organisation disintegrates.

Our results suggest that an abnormal persistence of cell adhesions is a key part of the Mpp5a and DNRab11a phenotypes and suggests that a lack of cell-cell separation might contribute to other epithelial disorganisation phenotypes in Crumbs- or Rab11a-deficient epithelia (Das and Knust, 2018; Roeth et al., 2009).

### Role of the C-division

A recent paper also concluded that Mpp5a is necessary to remodel junctional proteins during epithelialisation in the neural rod (Guo et al., 2018); however, these authors propose that a role of Mpp5a in apical remodelling is necessary only to rescue potential tissue disruption caused by midline C-divisions, a conclusion based on their reported rescue of lumen formation and apical junctional belts when cell division is blocked in the *mpp5a^m520^* mutant. However, we show that blocking cell division does not rescue junctional belt formation in *mpp5a^m227^* mutants (Fig. 6) and has a more fundamental role in apical junction remodelling than resolving cell connections during C-divisions.

We previously demonstrated that C-divisions at the neural rod midline are an important morphogenetic force during neural tube formation. Ectopic divisions are able to organise ectopic neural lumens and duplicate the neural tube (Tawk et al., 2007), whereas neural tubes generated in the absence of C-divisions are less efficient at resolving cell interdigitation across the midline and have a disorganised morphology (Buckley et al., 2013). Here, we show that connections between ipsilateral and contralateral cells are pulled into the cleavage furrow of C-divisions (Fig. 2). This ingression of neighbours into the cleavage furrow is reminiscent of neighbour ingression during mitoses in *Drosophila* epithelia (Herszterg et al., 2013). Invasion of neighbours into the cleavage furrow also occurs during chick gastrulation, where it can be regulated developmentally to promote or impede cell intercalation between daughter cells, hence contributing to the control of morphogenetic movements (Firmino et al., 2016). In zebrafish, we show that multiple contralateral cell contacts are promoted through ingression of neighbouring cell processes into the cleavage furrow. We propose that the maintenance of connections with multiple contralateral cells through C-divisions ensures that cells do not connect solely to their sisters and suggest that this is one mechanism leading to staggered connections across the midline. This staggered alignment of endfeet might generate a morphogenetic advantage by allowing attachment to a larger number of cells than a one-to-one ‘mirror adhesion’ and therefore helps to colocalise apical junctions from multiple neighbouring cells to the midline. The localisation of junctional proteins at cell corners might also be important for allowing flexibility of cell movement during tissue remodelling (Finegan et al., 2019).

It is also likely that actomyosin-generated forces at the neural rod midline will contribute to apical junction assembly. Both actin (Yang et al., 2009) and non-muscle myosin (Gutzman and Sive, 2010) accumulate at the developing apical plane, and there is substantial evidence that biomechanical forces play a role in adherens junction dynamics in other systems (Pinheiro and Bellaiche, 2018). Recent findings also show that actomyosin-mediated tension is necessary for assembling ZO-1 at tight junctions during zebrafish gastrulation (Schwayer et al., 2019). In addition to the role of microtubules (Buckley et al., 2013), it would therefore be interesting to determine the role of actomyosin mechanics in the assembly of apical junctions within the zebrafish neural rod.

## MATERIALS AND METHODS

### Embryo care

All embryos were collected, staged and cultured according to standard protocols (Kimmel et al., 1995). All procedures were carried out with UK Home Office approval and were subject to local Ethical Committee review.

### Generation of Pard3-EGFP transgenic line

To generate the transgenic zebrafish line expressing Pard3-EGFP with endogenous spatial and temporal expression, we used BAC recombineering. We replaced the stop codon of the pard3-003/ASIP transcript (von Trotha et al., 2006), in the BAC clone DKEY-71E21 (Source Bioscience). This BAC clone contains ∼71 kb upstream of the pard3-003/ASIP start codon and ∼30 kb downstream from the targeted stop codon. We followed the protocol of Bussmann and Schulte-Merker (2011), with some modifications. The original protocol leaves a kanamycin selection cassette in the final BAC construct. However, we found that this led to cell death and a lack of endogenous Pard3-EGFP expression. To remove the kanamycin selection cassette, we substituted two reagents with those from the recombineering protocol described by Sarov et al. (2006) and added an extra ‘flip out’ step to remove the kanamycin cassette. Specifically, we used the pRedFlp4 and R6k-EGFP-FRT-kanR-FRT cassettes from Sarov et al. (2006). The EGFP-FRT-kan-FRT targeting cassette was PCR amplified with 50 bp homology arms at either end, to target it to replace the pard3-003/ASIP stop codon during BAC recombineering: forward primer, 5′-CACAGAAGCAGAACGGACGCAATGGACACCCCTCCACTTCAGACAGGTAC**AGCTCAGGAGGTAGCGG-**3′; reverse primer, 5′-AATTGAGTTTCATGATAGAACTTTGTATTTCTGCAATTCTGAAAAGCTGA**GGCAGATCGTCAGTCAG**-3′. Nucleotides in bold amplify the EGFP-FRT-kan-FRT targeting cassette. The nucleotides in regular text are the 50 bp homology arms that target the cassette to replace the pard3-003/ASIP stop codon during BAC recombineering.

The protocols for pRedFlp4 transformation, targeted recombination steps and removal of the kanamycin resistance selection marker by pRedFlp4-induced flipase were carried out as previously described (Sarov et al., 2006), with slight modifications. Briefly, the recombineering pipeline was modified to have two rounds of tagging by Red/ET recombination; the first was insertion of iTol2_amp into the BAC backbone and the second insertion of EGFP-FRT-kanR-FRT. Successful recombination was assessed by colony PCR followed by full sequencing of the sites of recombination. Preparation and injection of BAC DNA and screening for transgenic founders was all performed according to standard protocols (Bussmann and Schulte-Merker, 2011; Suster et al., 2011).

### Zebrafish lines

The TgBAC(pard3:Pard3-EGFP)^kg301^ was generated as detailed above. The TgBAC(cdh2:Cdh2-tFT) (Revenu et al., 2014) was kindly provided by Darren Gilmour. The TgBAC(cdh2:Cdh2-tFT) line expresses both Cdh2-sfGFP and Cdh2-tagRFP, but we image only Cdh2-sfGFP expression in our experiments and refer to this as Cdh2-GFP for simplicity. These lines were bred with the Mpp5a^m227^ mutant fish line (Wei and Malicki, 2002) to generate compound Mpp5a^m227^;Cdh2-tFT and Mpp5a^m227^;Pard3-EGFP fish. To prevent ventricle opening in rhombomeres 3 and 5, we crossed the UAS (upstream activating sequence)-inducible dominant-negative Rab11a line, Tg(UAS:mCherry-Rab11a S25N)^mw35^ (Clark et al., 2011), with Tg(Egr2a:RFP-KalTA4), which drives the optimised Gal4-activator, KalTA4, only in Egr2a^+^ rhombomeres 3 and 5 (Distel et al., 2009). This resulted in the expression of Rab11a-S25N specifically in rhombomeres 3 and 5, as previously described (Buckley et al., 2013).

### RNA preparation and injection

Fusion constructs containing cDNA in the pCS2+ vector were linearised, and mRNA was synthesised using the SP6 mMessage mMachine kit (Ambion, AM1340). RNA for the following constructs was injected using standard protocols (Westerfield, 2000) at 40-100 pg per embryo: Human Histone 2A tagged with GFP (H2A-GFP), Human Histone 2B tagged with RFP (H2B-RFP), Human CAAX membrane moiety tagged with EGFP, mCherry or mNeptune2.5 (EGFP-CAAX, mCherry-CAAX or mNeptune2.5-CAAX), Dominant-negative Human rab protein 11a tagged with EGFP (RAB11A-S25N-EGFP) and Zebrafish partitioning-defective 3 tagged with GFP or RFP (Pard3-GFP/RFP). For ubiquitous distribution of mRNA, embryos were injected at the one- to two-cell stage. For mosaic labelling, a single blastomere of a 16- to 64-cell-stage embryo was injected.

### Morpholino injections

Co-injection of *mpp5a* splice blocking morpholinos (donor: 5′-GTTTATGACACCCACCTAGTAAAGC-3′ and acceptor: 5′-CTCCAGCTCTGAAAGTACAAACACA3′) was made into one-cell-stage embryos (Hsu et al., 2006). Full loss of Crb2a from the midline and associated phenotypes were seen with ∼1.7 nl of 300 mM (0.5 pM) of each morpholino, which closely phenocopied the *mpp5a* mutant phenotype (Fig. S3; Hsu et al., 2006). Mild to intermediate loss of Crb2a from the midline was seen with ∼0.5-1.0 nl of 200-300 mM (0.1-0.3 pM) of each morpholino. The level of Crb2a loss was correlated with the extent of associated phenotypes (see Fig. S3). The standard morpholino sequence was 5′-CCTCTTACCTCAGTTACAATTTATA-3′.

### *mpp5a* morphant and mutant comparison

In order to image cell behaviour in a Mpp5a-deficient background, we sometimes used *mpp5a* morpholino knockdown rather than *mpp5a* mutant embryos because we could not identify mutant embryos before imaging. We therefore confirmed that the phenotypes were comparable between *mpp5a* morphants and mutants (Fig. S3). We used a previously characterised morpholino (Hsu et al., 2006) and found that a morpholino concentration of 0.5 pM resulted in the loss of detectable Crb2a protein from the nascent apical surface and recapitulated the closed lumen phenotype of mutant embryos.

### Chemical inhibition of cell division

Cell division was blocked by incubating dechorionated embryos in 300 µM aphidicolin (Sigma) in 4% dimethyl sulfoxide (DMSO) from the bud stage (10 hpf) to the 28-somite stage (23 hpf). Control embryos were incubated in 4% DMSO only. Embryos were maintained on a bed of agarose in 12-well plates in the dark throughout the drug incubation.

### Immunohistochemistry

Embryos were fixed with 4% paraformaldehyde for 2 h at room temperature before processing for immunohistochemistry. The following primary antibodies were used: mouse anti-Crb2a (mouse monoclonal, ZIRC, zs-4, 1:200), rabbit anti-Crb2a and anti-Crb1 (a kind gift from the Wei laboratory, 1:350), mouse anti-ZO-1 (mouse monoclonal, Invitrogen, 33-9100, 1:500), rabbit anti-aPKC (rabbit polyclonal, Santa Cruz, sc-216, 1:300), mouse anti-gamma-tubulin (mouse monoclonal, MilliporeSigma, T6557, 1:200) and mouse anti-ZO-1 (Invitrogen, 33-910, 1:300). Rabbit anti-Pard3 (rabbit polyclonal, Millipore, 07-330, 1:100) was used after fixation in Dent's fixative for 3 h at room temperature and rehydration from methanol. Alexa Fluor secondary antibodies (Life Technologies) were used at 1:500, and Hoechst (Life Technologies) nuclei stain was used at a final concentration of 1:10,000.

### Electron microscopy

Embryos were fixed in 2% paraformaldehyde and 1.5% glutaraldehyde in 0.1 M sodium cacodylate buffer overnight and then processed through 1% osmium and stained in 0.5% uranyl acetate. They were then dehydrated in ethanol and processed through propylene oxide into 100% resin, embedded and baked. Ultra-thin sections were taken in transverse orientation and imaged in a transmission electron microscope.

### Confocal imaging and processing

Embryos were mounted in low-melting-point agarose and imaged using a Zeiss LSM 880 Fast Airyscan microscope and water-dipping ×20/1.0 NA objective, a Leica SP5 confocal microscope and water-dipping ×25/0.95 NA objective or a PerkinElmer Ultraview spinning disc microscope. Imaging at the centre of a developing live tissue mass required the improved resolution in *x*, *y* and *z* coordinates obtained by using the Fast Airyscan mode of the Zeiss LSM 880 Fast Airyscan microscope. By using this microscope, high-resolution images of both *en face* Pard3-EGFP rings and the spatial location of Crb2a, aPKC and Cdh2-EGFP in the developing embryo were obtained. To improve lateral imaging, fish were mounted on an agarose bed with small depressions to hold the yolk of the embryo in place. These agarose beds were made using a custom mould (a kind gift from Andrew Oates). Data were collected from the hindbrain and anterior spinal cord regions. In Fig. 4F, some overlying cells have been manually edited from image to increase clarity of the cells of interest. Unedited versions of these images can be seen in Fig. S1. Images were processed using Imaris, Volocity and Fiji/ImageJ.

### Data analysis

The custom R code used to generate plots for Figs 1 and 3 is available at https://github.com/andyivanhoe/pard3-analysis.

To obtain apical ring density counts across the developing neuroepithelium (Fig. 1E), Pard3-EGFP embryos were live-imaged *en face* between the 15- and 21-somite stages using the Zeiss Airyscan. The dorsoventral axis of the resulting *z*-stacks was divided into four quadrants (dorsal, mid-dorsal, mid-ventral and ventral), by applying a grid in Fiji. Apical rings were counted when all sides of the cell border had Pard3-EGFP signal along them.

To quantify the changing distribution of Pard3-EGFP across the neural rod midline over time, we imaged six live TgBAC(pard3:Pard3-EGFP)^kg301^ embryos over a period of 3 h using Zeiss Airyscan confocal acquisition and processing. We then made a maximum-intensity projection of a 10-µm-deep volume at a mid-dorsoventral level of the neural rod. A mediolateral line of 116 µm width was then drawn across the basal-to-basal width of the developing neuroepithelium and the mean fluorescence intensity at each pixel along that line measured using Fiji. This was repeated for six embryos at 1 h intervals (Fig. 3Aii,Aiii; 0 h is 16-somite stage). All line profiles from different embryos and time points were aligned on the maximum intensity value for each line profile. The central 70 µm of the line profiles, centred on the maximum intensity values, were then used to calculate the mean intensity and standard deviation at each point along the line for each time point before plotting. We aligned on the maximum intensity value for each line profile because the exact maximum intensity of Pard3-EGFP relative to the tissue midline exhibits slight biological variation between embryos.

To quantify the distribution of Pard3-EGFP at the midline at the fully neuroepithelial stage (after the single midline peak had resolved into what appeared to be two near-parallel lines of expression on either side of the midline), we analysed a single *z*-level in the same six embryos where the two lines of expression were ∼4 µm apart (Fig. 3Aiv). This analysis was done at the last time point of the 3 h timeline, but at a more ventral (hence, more morphogenetically advanced; see Fig. 1 and associated text) position. In this case, we measured intensity along a 23-µm-wide mediolateral line. Given that the midline expression was not perfectly straight and the peaks of expression were only 4 µm apart, for this analysis we chose a single *z*-level and a thinner mediolateral line than for the timeline measurements to avoid averaging out the two peaks.

Plots in Figs 4C,J and 7G,H were generated using Graphpad Prism. Specific statistical analyses are described in the figure legends.

## Supplementary Material

Supplementary information

Reviewer comments
