# Supplementary material for: Coordinated assembly and release of adhesions builds apical junctional belts during de novo polarisation of an epithelial tube
Source: Development. 2020 Dec 23;147(24):dev191494. doi: 10.1242/dev.191494 (PMC7774892; doi:10.1242/dev.191494)
Supplement: Supplementary information [file develop-147-191494-s1.pdf]

Figure S1. Image editing in Figure 4F

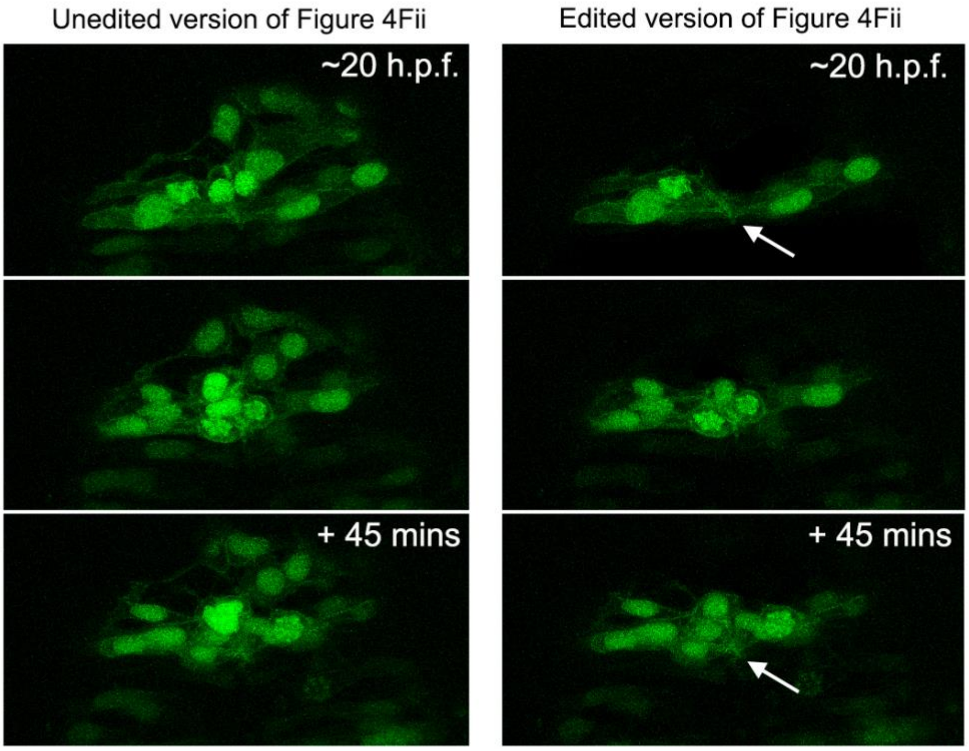

Side-by-side comparisons of edited and unedited versions of Figure 4Fii. All panels are maximum intensity projection images from an 11 µm deep z-stack. In the unedited version, some cells that do not contribute to the rosette organisation obscure the clarity of the rosette formation. These obscuring cells were manually edited out of the relevant z-levels to generate the images shown in the edited version. Arrows in the edited version indicate centre of rosette where many cell processes seem to meet.

Development • Supplementary information

Figure S2. *mpp5a^{m227}* mutants still open a lumen at the midbrain-hindbrain boundary

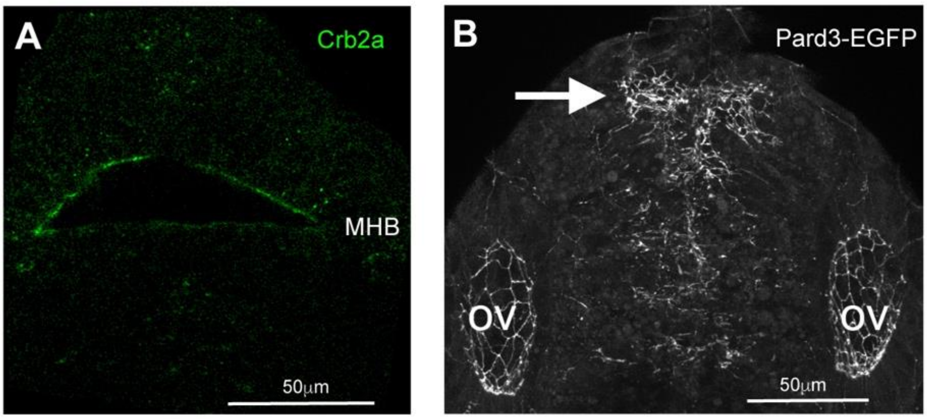

A. Single horizontal z-plane of the hindbrain neural rod from a *mpp5a^{m227}* mutant embryo at the level of the midbrain-hindbrain boundary (MHB), imaged for Crb2a immunoreactivity. A lumen has opened, which is lined with Crumbs2a protein apically (5/5 embryos).
B. A 57 µm maximum projection of a z-stack of the hindbrain neural rod from a 28 somite stage *mpp5a^{m227}* mutant; Pard3-EGFP compound transgenic embryo at the level of the otocysts. Apical rings have formed at the lumen surface (arrow, 4/4 embryos).

Development • Supplementary information

Figure S3: *mpp5a* morphant and mutant phenotypes

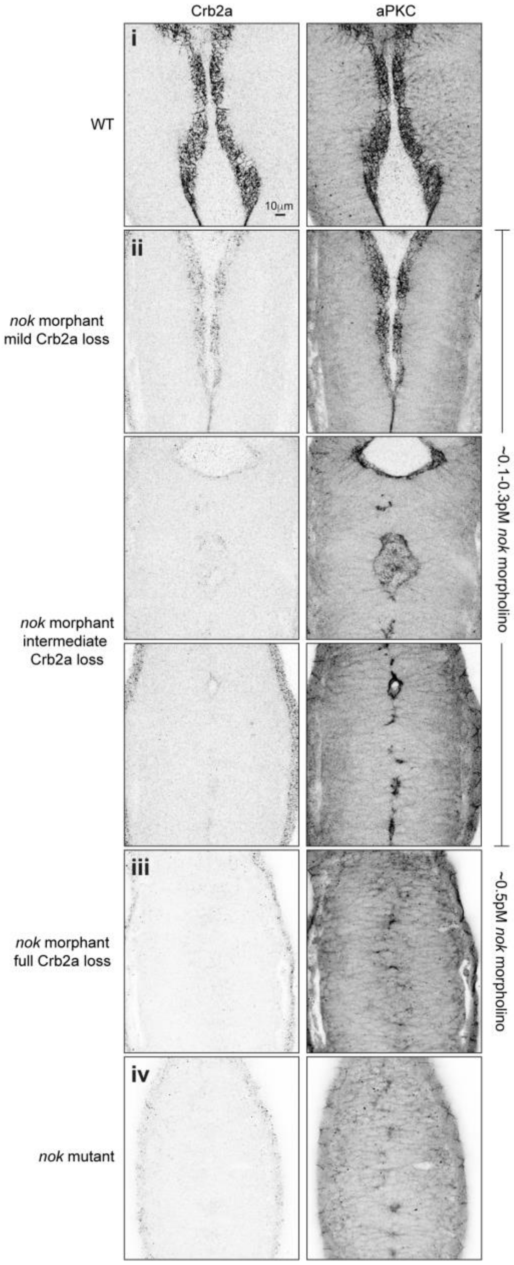

Dorsal views of 10µm Z-projections at a mid dorso-ventral level through the hindbrains of 30 h.p.f. embryos, stained via IHC for Crb2a and aPKC **i)** Wild-type embryos had open lumens lined apically with extensive Crb2a and aPKC protein (5/5 embryos). **ii)** In early *mpp5a* morphant experiments, a lower concentration range of *mpp5a* morpholinos was tested (approximately 0.1-0.3pM). This resulted in mild (17/21 embryos) or intermediate (11/21 embryos) loss of Crb2a from the tissue midline. The level of Crb2a loss correlated with the extent of associated phenotypes (lack of lumen opening, disorganised midline and ectopic apical proteins). **iii)** In later *mpp5a* morphant experiments, a higher concentration of *mpp5a* morpholinos was used (approximately 0.5pM). This resulted in an almost full or full loss of Crb2a from the posterior hindbrain tissue midline (3/3 embryos), a closed lumen, disorganised midline and ectopic apical proteins (6/8 embryos, see figure 4D). **iv)** *mpp5a* mutant embryos phenocopied the later *mpp5a* morpholino experiments, also showing a full loss of Crb2a from the posterior hindbrain tissue midline (4/4 embryos), a closed lumen, disorganised midline and ectopic apical proteins (9/9 embryos, see figure 4D).

Development • Supplementary information

Movie 1 accompanies Figure 1G.

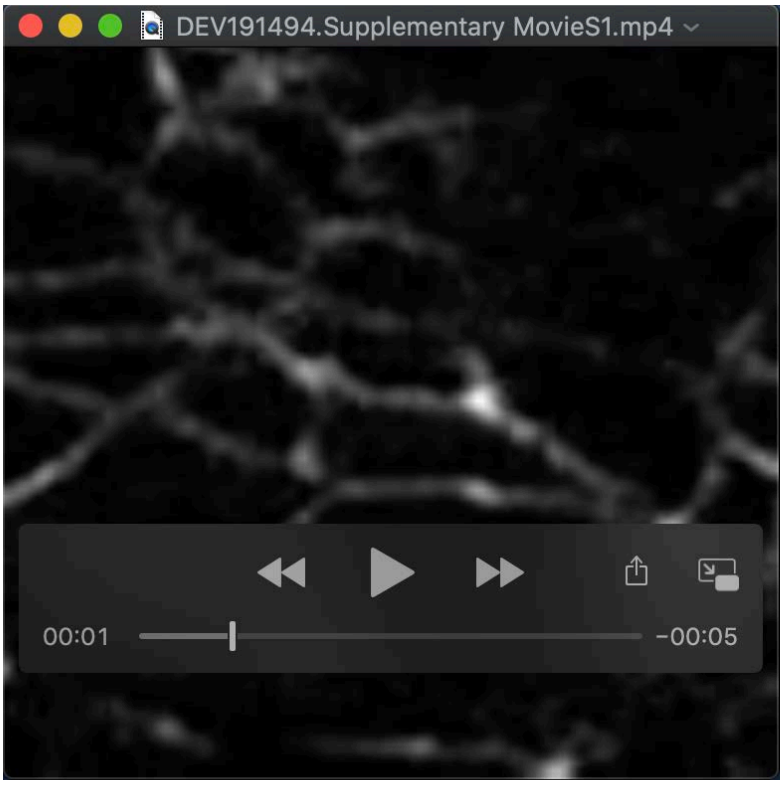

Movie 1: This movie shows an example of the typical offset arrangement of apical rings either side of the tissue midline, prior to lumen opening. A Pard3-EGFP embryo was live-imaged *en-face* to the developing apical surface plane at 18-somite stage. The movie follows the z-stack from one side of the tissue midline, along the medio-lateral axis of the embryo, through the tissue midline to the other side of the embryo in 0.31 μm z-steps.

Movies 2-4 accompany Figure 2A.

Collectively, these movies demonstrate that the shape of the nascent apical endfeet (and therefore the location of the connection point between sister cells in relation to the rest of the cell shape) dynamically alters over the neural keel to rod transition. This is likely in response to the movements and mitoses of neighbouring (unlabelled) cells.

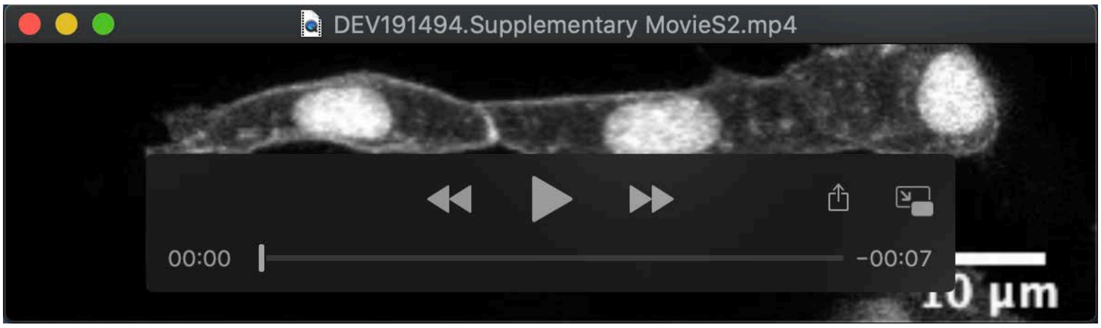

Movie 2: 8-minute-long movie illustrating the connection between sister cells from the 13-somite stage. The nascent apical endfoot of the left-hand cell transiently moves posteriorly during the movie.

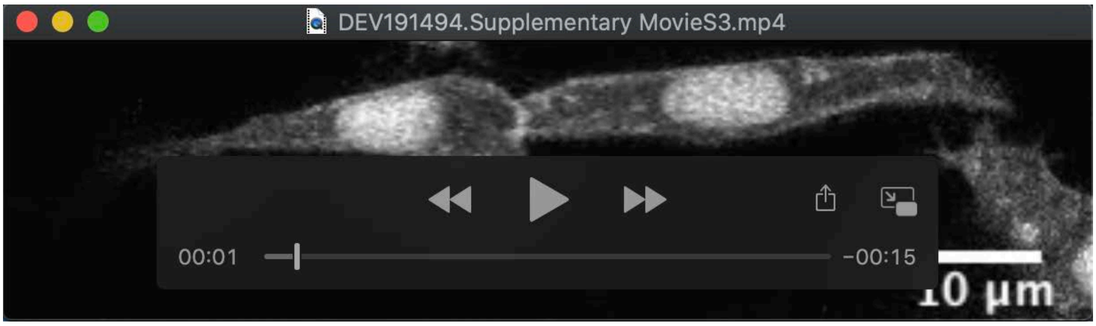

Movie 3: 19-minute-long movie illustrating the connection between sister cells from the 14-somite stage. The connection point between sister cells is initially narrower than in the previous movie and then widens again to occupy the whole nascent apical endfeet of both cells.

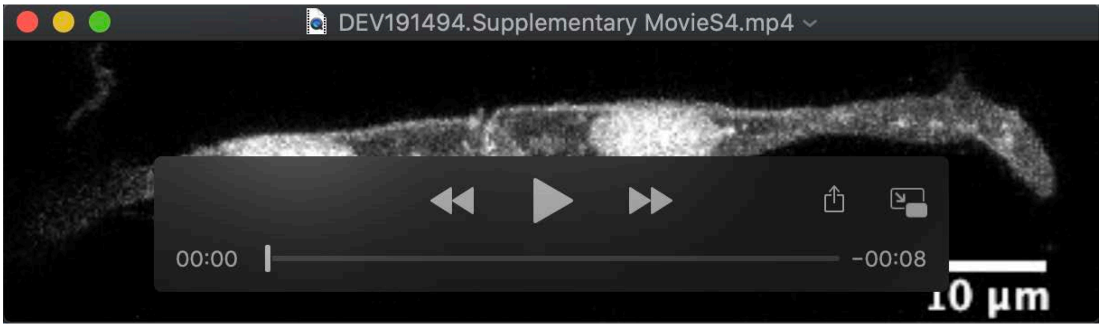

Movie 4: 10-minute-long movie illustrating the connection between sister cells from the 15-somite stage. The nascent apical endfoot of the left-hand cell narrows and the relative position of its connection point with the right hand cell moves first to the anterior side and then to the posterior side of the right hand cell.

Development • Supplementary information
