# Supplementary material for: Coordinated assembly and release of adhesions builds apical junctional belts during de novo polarisation of an epithelial tube
Source: Development. 2020 Dec 23;147(24):dev191494. doi: 10.1242/dev.191494 (PMC7774892; doi:10.1242/dev.191494)
Supplement: Reviewer comments [file develop-147-191494_review_history.pdf]

Coordinated assembly and release of adhesions builds apical junctional belts during de novo polarisation of an epithelial tube

Andrew C. Symonds, Clare E. Buckley, Charlotte A. Williams and Jonathan D. W. Clarke
DOI: 10.1242/dev.191494

Editor: Stephen Wilson

Review timeline
Original submission: 9 April 2020
Editorial decision: 7 May 2020
First revision received: 21 September 2020
Editorial decision: 9 October 2020
Second revision received: 27 October 2020
Accepted: 28 October 2020

Original submission

First decision letter

MS ID#: DEVELOP/2020/191494

MS TITLE: Coordinated assembly and release of adhesions builds apical junctional belts during de novo polarisation of an epithelial tube

AUTHORS: Andrew C Symonds, Clare E Buckley, Charlotte A Williams, and Jonathan DW Clarke

I have now received all the referees' reports on the above manuscript, and have reached a decision. The referees' comments are appended below, or you can access them online: please go to BenchPress and click on the 'Manuscripts with Decisions' queue in the Author Area.

As you will see, the referees like your study, but have some suggestions for improvements before publication. The comments from reviewers one and two should be relatively straightforward to address whereas Reviewer 3 would ideally like more experimental data. However given the current difficulty in performing experiments, I appreciate that you may not wish to delay publication to do more benchwork. If you consider that the suggested experiments are not essential for this study, then you should explain this in your response to the reviewers comments. I should also add that neither I nor the other two reviewers are concerned about the level of advance over the Guo et al. paper.

Please attend to all of the reviewers' comments and ensure that you clearly highlight all changes made in the revised manuscript. Please avoid using 'Tracked changes' in Word files as these are lost in PDF conversion. I should be grateful if you would also provide a point-by-point response detailing how you have dealt with the points raised by the reviewers in the 'Response to Reviewers' box. If you do not agree with any of their criticisms or suggestions please explain clearly why this is so.

© 2020. Published by The Company of Biologists under the terms of the Creative Commons Attribution License (https://creativecommons.org/licenses/by/4.0/).

Reviewer 1

Advance summary and potential significance to field

This manuscript describes the processes by which a ball of cells create a continuous lumen de novo during zebrafish neurogenesis. The authors distill this process into three phases: spot-to-ring junction building, displacement of apical determinants from the apical-most surface to an apicolateral junction belt, cell division across the nascent interface. I am not aware of any other work that visualises and describes this important sequence as beautifully. The comments below should be addressed to refine the manuscript without requiring additional experimentation.

Comments for the author

1) The manuscript is beautifully illustrated with very convincing micrographs. Quantification would give the reader greater confidence in reproducibility and effect magnitude. For example, in figure 3ai it would be meaningful to compare the ratio of fluorescence intensity between vertical cell borders and horizontal borders at the neural midline and compare this ratio between embryos. This intensity quantification would then also be relevant to Figure 7.
2) The process of Pard3 coalescence is not obvious form the images provided. Figure 1 B and E appear to show graded fluorescence intensity which increases across developmental time and AP position. The distinct distributions described as puncta versus rings are very clear, but the images provided seem to show a binary pattern of either puncta or rings. Can the authors replace the single image in 1d with a series showing cells at different stages of the puncta to ring transition?
3) "…developing rings on either side of the tissue midline are always offset from one another, rather than having a mirror symmetric arrangement that might be expected if individual rings split into two." While this seems a sensible assertion, it is likely that local differences in cell packing would produce asymmetry. Can the authors provide LHS/middle/RHS images of a larger field of view and perform rigid body image registration to give a clearer picture of irreconcilable differences in symmetry?
4) The presence of hydroxyurea with aphidicolin in the cell cycle inhibition experiment is only apparent is one reads the methods carefully as if reviewing the manuscript. Please refer to use of this compound in the results and figure 7.
5) References to "enlarged size of the apical rings" and "enlarged nuclear size" suggests quantification. Please provide these quantifications.
The presentative image showing redistribution of nuclei away from the midline in 7G (ignoring the abundant apoptotic bodies) is also very suggestive of an S phase block in a pseudostratified epithelium.
6) Page 6 line 12 – the description of "mediolaterally oriented stripes" makes one think of Drosophila segment polarity stripes. Please rephrase.
7) Given the recent high-profile paper from CP Heisenberg's group
(Cell, 2019) showing mechanical tension-related F-actin flows assemble apical tight junctions, can the authors comment on the roles of the cytoskeleton in assembling apical junctions and particularly in the redistribution of Zo-1? The Guo et al iScience paper the authors refer to shows changes in F-actin distribution which are mirrors the Pard3 and Cdh2 distributions shown here. Both the F-actin and microtubule cytoskeletons are likely to be critical in localisation of apical determinants and both these authors and others have published extensively on the subject.

Reviewer 2

Advance summary and potential significance to field

This is a carefully written paper which presents a series of well designed and executed experiments elucidating the roles of key proteins in neuroepithelial cell remodelling as the zebrafish neural keel creates a lumen and transforms into a neural tube. The work takes advantage of zebrafish reporter lines, live imaging and EM to provide detailed and high-quality data describing cell re-arrangements that precede lumen formation and accompanying localisation dynamics of cell biological proteins known to regulate cell adhesion and junction formation. The authors identify two essential steps, release of contralateral cell-cell adhesions across the midline and formation of apical belts along the newly emerging apical edge as critical events in lumen formation. Using morpholinos and a

© 2020. Published by The Company of Biologists under the terms of the Creative Commons Attribution License (https://creativecommons.org/licenses/by/4.0/).

dominant negative construct, they uncover roles for proteins Nok and Rab11a in the regulation of a required transmembrane protein Crumbs in these processes. Finally, they additionally clarify an earlier report demonstrating the requirement for Nok for apical ring formation, even in the absence of cell division.

Overall, the paper provides important new insights into the molecular underpinnings of this form of neurulation. Some revisions, including further experimental details and discussion are proposed below.

Comments for the author

Comments

1) Please provide numbers of biological replicates / embryos imaged and analysed for all descriptive data (including EM) presented in figures 1,2 and 3 (appreciate that some information is in the text e.g. Figures 1F, G, and fig 2A in legend). Apologies if I have missed this - is there a minimal number of embryos in which observations are made in all cases?

2) Numbers of biological replicates for experiments in Figure 4 are erratically provided in the figure legend. For example, is the graph in 4C just for cells in one embryo? Numbers of experiments and embryos using morpholinos and reproducibility of these observations are important. Information about scrabbled? control morpholinos (in methods) and their analysis is also lacking, data in Figure 4 show comparison of WT vs morphant, why not morpholino control?

3) Overall, it would be helpful if numbers of exps and embryos were provided consistently for all experiments in all figure legends.

4) In the Discussion, other contexts in which Crumbs regulates apical adhesion in vertebrate neural tube, should be discussed, for example, recent work demonstrating a role for Crumbs in the regulation of neuroepithelial cell delamination during remodelling of the mouse spinal cord central canal Tait et al 2020 PMID:32150534. It might be interesting to discuss this in light of their finding that non cell autonomous rescue by wildtype cells is able to initiate epithelialisation in the absence of Rab11a/Crumbs.

5) The Discussion ends abruptly and lacks concluding sentences that place these findings in a wider context. It would be useful for example to make comparisons with what is known of molecular mechanisms regulating secondary neurulation in the caudal regions of chick and mouse embryos.

Reviewer 3

Advance summary and potential significance to field

Please refer to the uploaded document

Comments for the author

The manuscript entitled “Coordinated assembly and release of adhesions builds apical junctional belts during de novo polarisation of an epithelial tube” by Symonds et al uncovers the mechanism underlying polarization of neuroepithelial cells and the importance of its modulation in the formation of the neural tube. To begin with, the authors describe the offset arrangement of the neuroepithelial cells on the two sides of the tube and the process of de-novo polarity establishment. They also show evidence that this polarity establishment requires the function of *nagie oko*. Going further, the authors elucidate that the contralateral adhesion between the cells is resolved while the formation of ipsilateral junctions is promoted which further facilitates the lumen formation. This process is regulated by *nagie oko* and Rab11.
The authors propose that Nok and Rab11 operate via Crumbs during this remodeling of the junctions.

© 2020. Published by The Company of Biologists under the terms of the Creative Commons Attribution License (https://creativecommons.org/licenses/by/4.0/).

Major Comment

The authors have studied an important aspect of de novo polarity establishment, its modulation, and its subsequent link with the lumen formation. This is a very well‐conducted investigation. The conclusions drawn are based on solid experimental evidence that are obtained by carefully performed experiments involving high‐end live imaging. It is rather unfortunate that a similar study has already been published by Guo et at (iScience 3, 63–85, May 25, 2018). As a result, in its current form, the manuscript offers minimal conceptual advance, which is limited to showing that the epithelial cells are organized offset at the midline and that Nok dependent apical resolution is required in both dividing and non‐dividing cells. Unfortunately, the importance of Crb2a in this process – which could have hugely enhanced the significance of the study ‐‐‐ remains understudied. Neither the Nok loss of function nor DN Rab11 directly implicates Crumbs in the process of junctional remodeling. In fact, DN Rab11 is expected to have a broader effect on cell polarity and the results obtained should be interpreted carefully. While it is true that the functional redundancy may make the genetic analysis difficult for Crb, authors could possibly try other ways such as dominant‐negative versions of Crb and show it's a mechanistic link with junctional remodeling. Another aspect that could be further explored is whether the steps involved in junctional modeling and the underlying mechanism remains the same or changes in the absence of cell division. From the current analysis, it is not clear whether the contralateral cells are organized in an offset manner in the absence of proliferation and whether Crb2a‐Nok‐Rab11 dependent cell adhesion modulation works exactly the same way as shown for the proliferating neuroepithelial cells.

Minor comments

1. Using vertices instead of multicellular interfaces would be more appropriate.
2. Authors state that "However, following the C‐division the interface between sister cells was highly dynamic. Both the shape and the location of the sister interface changed over time" This is not convincing from the still images. It will be good to include movies.
3. Authors show that Cdh2 and Pard3 are cleared off from the apical domain and relocalise to the apicolaterally. This analysis needs to be performed in the presence of a membrane marker or any other apical marker to get the sense of clearing off and relocalisation.
4. Cell proliferation needs to be directly assessed in Aphidicolin treated embryos.
5. (Figure 3Bi) and (Figure 3Bii) – in the figures i and ii labels are missing.
6. Given the wealth of literature on cell polarity and junctional remodeling, the introduction reads too thin.

First revision

<u>Author response to reviewers' comments</u>

General Comment. We thank the reviewers for their helpful comments and we have rewritten many passages of our manuscript to try to increase clarity, reduce speculation and add precision. We have also included new data and quantification and modified many aspects of our Figures to illustrate these modifications. We think our manuscript is now greatly improved.

Point by point replies to Reviewer comments:

Reviewer 1:
1) The manuscript is beautifully illustrated with very convincing micrographs. Quantification would give the reader greater confidence in reproducibility and effect magnitude. For example, in figure 3ai it would be meaningful to compare the ratio of fluorescence intensity between vertical cell borders and horizontal borders at the neural midline and compare this ratio between embryos. This intensity quantification would then also be relevant to Figure 7.

© 2020. Published by The Company of Biologists under the terms of the Creative Commons Attribution License (https://creativecommons.org/licenses/by/4.0/).

We have now included a quantification in figure 3 of mean Pard3 intensity across the basal-to-basal width of the developing neuroepithelium over time, which shows that the midline expression domain of Pard3 increases in intensity and decreases in width over neural rod development. We also include an example of the fluorescence intensity of the parallel ‘tram-line’ of Pard3 seen at fully epithelial stages. We can’t compare the ratio of fluorescence intensity between vertical cell borders and horizontal borders at the neural midline since the cells are interdigitated at early stages and so don’t have horizontal borders until they are fully epithelialized. The main message from figure 7 is that nok mutants do not make apical rings, even in the absence of cell division (compared with wild types in figure 1B) so we have not included a similar analysis for figure 7.

2) The process of Pard3 coalescence is not obvious form the images provided. Figure 1 B and E appear to show graded fluorescence intensity which increases across developmental time and AP position. The distinct distributions described as puncta versus rings are very clear, but the images provided seem to show a binary pattern of either puncta or rings. Can the authors replace the single image in 1d with a series showing cells at different stages of the puncta to ring transition?

We have replaced the image in figure 1D and have changed the wording in the results section to make the Pard3 coalescence clearer. We have also quantified the formation of mature Pard3 apical rings over developmental time and dorsoventral position (figure 1E).

3) “…developing rings on either side of the tissue midline are always offset from one another, rather than having a mirror symmetric arrangement that might be expected if individual rings split into two.” While this seems a sensible assertion, it is likely that local differences in cell packing would produce asymmetry. Can the authors provide LHS/middle/RHS images of a larger field of view and perform rigid body image registration to give a clearer picture of irreconcilable differences in symmetry?

We have included a movie of the z-stack for this data (supplementary movie 1), taken from a wider field of view. It is not possible to carry out a rigid body image registration since each forming ring is at a slightly different angle to the midline of the tissue. We fully agree that ‘local differences in cell packing would produce asymmetry’. We believe that these asymmetries arise during an earlier stage of development and result from the movement of surrounding cells that remain attached at the cleavage furrow of dividing neuroepithelial progenitor cells. This forms the basis of our conclusions from figure 2. So, in summary, local cell movements following C-division leads to an offset between sister cells, which then later independently form apical rings on either side of the midline. We have clarified our model in figure 2Biii and the text describing figure 2 to make this time-line clearer. We have also included movies to accompany figure 2A (supplementary movies 2-4), so that the dynamic nature of the nascent apical endfeet of sister cells can be seen more clearly.

4) The presence of hydroxyurea with aphidicolin in the cell cycle inhibition experiment is only apparent is one reads the methods carefully as if reviewing the manuscript. Please refer to use of this compound in the results and figure 7.

Thank you for pointing this out - this was an error in our methods since we only used aphidicolin. We have amended our methods and text accordingly.

5) References to “enlarged size of the apical rings” and “enlarged nuclear size” suggests quantification. Please provide these quantifications.

We have added quantifications of nuclear size and numbers of apical rings per area to figure 7, which demonstrate the efficiency of the S-phase division block.

6) Page 6 line 12 – the description of “mediolaterally oriented stripes” makes one think of Drosophila segment polarity stripes. Please rephrase.

This sentence now reads: “At the 11-somite stage (14 h.p.f., neural keel stage) puncta of Pard3-EGFP were localised broadly around the tissue midline, along the interfaces of interdigitating cells.”

© 2020. Published by The Company of Biologists under the terms of the Creative Commons Attribution License (https://creativecommons.org/licenses/by/4.0/).

Reviewer 2:
1) Please provide numbers of biological replicates / embryos imaged and analysed for all descriptive data (including EM) presented in figures 1,2 and 3 (appreciate that some information is in the text e.g. Figures 1F, G, and fig 2A in legend). Apologies if I have missed this - is there a minimal number of embryos in which observations are made in all cases?

We think we have now supplied all the n numbers for our observations and more completely quantified the data (see reply to Reviewer 1 Point 1).

2) Numbers of biological replicates for experiments in Figure 4 are erratically provided in the figure legend. For example, is the graph in 4C just for cells in one embryo? Numbers of experiments and embryos using morpholinos and reproducibility of these observations are important. Information about scrabbled? control morpholinos (in methods) and their analysis is also lacking, data in Figure 4 show comparison of WT vs morphant, why not morpholino control?

We have extended the analysis in Figure 4C to include division locations from 3 nok morphant embryos. We have included division location data from 1 wild type embryo as a reference. However, wild type divisions always occur exactly at the tissue midline or lumen surface, except for rare basal divisions, as referenced in the figure legend. We do not have equivalent data from control morpholino injected embryos for this experiment. However, we did validate that nok morphants and mutants give the same phenotypes and that the level of Crumbs knock down in morphants correlates with the extent of phenotypes seen (data now included in supplementary figure 1). We did use standard control morpholino to validate some of our experiments (e.g. we know that apical ring formation was not altered in control morphants and have now included these numbers in figure legend 1E). The sequence for the standard control morpholino is now included in the methods section.

3) Overall, it would be helpful if numbers of exps and embryos were provided consistently for all experiments in all figure legends.

We have now done this.

4) In the Discussion, other contexts in which Crumbs regulates apical adhesion in vertebrate neural tube, should be discussed, for example, recent work demonstrating a role for Crumbs in the regulation of neuroepithelial cell delamination during remodelling of the mouse spinal cord central canal Tait et al 2020 PMID:32150534. It might be interesting to discuss this in light of their finding that non cell autonomous rescue by wildtype cells is able to initiate epithelialisation in the absence of Rab11a/Crumbs.

We are happy to include this interesting paper in our discussion, which we read just after submitting our manuscript.

5) The Discussion ends abruptly and lacks concluding sentences that place these findings in a wider context. It would be useful for example to make comparisons with what is known of molecular mechanisms regulating secondary neurulation in the caudal regions of chick and mouse embryos.

We have briefly mentioned that it would indeed be interesting to know whether similar mechanisms operate during secondary neurulation in birds and mammals. The cellular and molecular details of lumen formation during secondary neurulation in these animals is rather shockingly limited so there is nothing really to compare our data to in this respect.

Reviewer 3:
Major Comment
The authors have studied an important aspect of de novo polarity establishment, its modulation, and its subsequent link with the lumen formation. This is a very well- conducted investigation. The conclusions drawn are based on solid experimental evidence that are obtained by carefully performed experiments involving high-end live imaging. It is rather unfortunate that a similar study has already been published by Guo et at (iScience 3, 63-85, May 25, 2018). As a result, in its current

© 2020. Published by The Company of Biologists under the terms of the Creative Commons Attribution License (https://creativecommons.org/licenses/by/4.0/).

form, the manuscript offers minimal conceptual advance, which is limited to showing that the epithelial cells are organized offset at the midline and that Nok dependent apical resolution is required in both dividing and non-dividing cells. Unfortunately, the importance of Crb2a in this process – which could have hugely enhanced the significance of the study - remains understudied. Neither the Nok loss of function nor DN Rab11 directly implicates Crumbs in the process of junctional remodeling. In fact, DN Rab11 is expected to have a broader effect on cell polarity and the results obtained should be interpreted carefully. While it is true that the functional redundancy may make the genetic analysis difficult for Crb, authors could possibly try other ways such as dominant-negative versions of Crb and show it's a mechanistic link with junctional remodeling. Another aspect that could be further explored is whether the steps involved in junctional modeling and the underlying mechanism remains the same or changes in the absence of cell division. From the current analysis, it is not clear whether the contralateral cells are organized in an offset manner in the absence of proliferation and whether Crb2a-Nok-Rab11 dependent cell adhesion modulation works exactly the same way as shown for the proliferating neuroepithelial cells.

We are unable to explore the role of Crumbs more directly at the moment due to the Covid restrictions, but we agree we had probably overstated our results in relation to Crumbs function. We have moderated all references to Crumbs function to more clearly state our data tests the function of nok and Rab11a. We still prefer the hypothesis that these likely operate through their deployment of Crumbs but hopefully this is now more clearly reasonable speculation rather than tested hypothesis.

We agree it would be interesting to know whether the steps we uncover remain the same in the absence of division, but this was not the aim of our current and with respect we decline to enter into a whole new aspect of the study. Since the fish neural rod does use divisions to help orchestrate the development of its nascent apical surfaces we think it is more important to understand how this occurs in its normal mode of development.

1) Using vertices instead of multicellular interfaces would be more appropriate.

Thank you for the suggestion – we have made this change

2) Authors state that "However, following the C-division the interface between sister cells was highly dynamic. Both the shape and the location of the sister interface changed over time" This is not convincing from the still images. It will be good to include movies.

We have included movies accompanying figure 2A (supplementary movies 2-4). These illustrate the dynamic nature of the nascent apical endfeet of sister cells at the 13, 14 and 15 somite stage respectively. We have also clarified the text describing this data. The key point here is that the C-division sisters became offset from each other by the neural rod stage.

3) Authors show that Cdh2 and Pard3 are cleared off from the apical domain and relocalise to the apicolaterally. This analysis needs to be performed in the presence of a membrane marker or any other apical marker to get the sense of clearing off and relocalisation.

We have now included a panel of 24 h.p.f. neuroepithelial cells in figure 3Avi, which shows that plasma membranes meet at the tissue midline while Pard3 is now largely located in two parallel parasagittal domains.

4) Cell proliferation needs to be directly assessed in Aphidicolin treated embryos.

We have added quantifications of nuclear size and numbers of apical rings per area to figure 7, which demonstrate the efficiency of the S-phase division block.

5) (Figure 3Bi) and (Figure 3Bii) – in the figures i and ii labels are missing.

Thank you for pointing this out. We have corrected this.

6) Given the wealth of literature on cell polarity and junctional remodeling, the introduction reads too thin.

© 2020. Published by The Company of Biologists under the terms of the Creative Commons Attribution License (https://creativecommons.org/licenses/by/4.0/).

We prefer our Introduction to be brief and to the point but we agree we were a bit minimal. We have rewritten the Introduction to include some reviews of epithelialisation and to better allude to the most relevant data on the remodelling of polarity and adhesion proteins in the generation of apico-lateral junctional belts. We are also at the manuscript's word limit, so this doesn't allow us to go into more detail.

Second decision letter

MS ID#: DEVELOP/2020/191494

MS TITLE: Coordinated assembly and release of adhesions builds apical junctional belts during de novo polarisation of an epithelial tube

AUTHORS: Andrew C Symonds, Clare E Buckley, Charlotte A Williams, and Jonathan DW Clarke

The referees are happy with your revisions and there are just a couple of minor points to consider before publication. The referees' comments are appended below, or you can access them online: please go to BenchPress and click on the 'Manuscripts with Decisions' queue in the Author Area.

Please attend to all of the reviewers' comments in your revised manuscript and detail them in your point-by-point response. If you do not agree with any of their criticisms or suggestions explain clearly why this is so.

Reviewer 1

Advance summary and potential significance to field

This manuscript demonstrates how a solid organ forms parallel polarised epithelial sheets with opposed apical surfaces by releasing contarlateral adhesions to form ipsilateral junction belts. This process requires the apical determinants NOK/Pals1/Mpp5a and Rab11a, likely acting through Crumbs.

Comments for the author

The authors have provided new data, quantification and clarifications which address my original concerns.

I am not able to open supplementary movies 2-4. These are not critical to my review but please make sure they are provided in an accessible format. Movie 1 opens with standard software.

"mouse anti-©tubulin" in the methods should probably read "gamma-tubulin".

The authors appear to have disregarded the 7th point in my original review which was only a minor point for discussion. It remains relevant:
Given the recent high-profile paper from CP Heisenberg's group (Cell, 2019) showing mechanical tension-related F-actin flows assemble apical tight junctions, can the authors comment on the roles of the cytoskeleton in assembling apical junctions and particularly in the redistribution of Zo-1? The Guo et al iScience paper the authors refer to shows changes in F-actin distribution which mirrors the Pard3 and Cdh2 distributions shown here. Both the F-actin and microtubule cytoskeletons are likely to be critical in localisation of apical determinants and both these authors and others have published extensively on the subject.

© 2020. Published by The Company of Biologists under the terms of the Creative Commons Attribution License (https://creativecommons.org/licenses/by/4.0/).

Reviewer 2

Advance summary and potential significance to field

This is a second review following revision.

Comments for the author

The authors have addressed the main concerns of this reviewer, although it would have been preferable to have used a scrabbled morpholino control it is understandable that this additional control cannot be undertaken in the current circumstances. The authors have also elaborated and deepened their discussion of related studies. Most importantly, the numbers of experiments and embryos are now provided consistently for all experiments in the figure legends. With further quantifications and these indications of reproducibility the paper is appropriate for publication.

Reviewer 3

Advance summary and potential significance to field

Not applicable as this is a revised version

Comments for the author

The authors have taken care of most of my comments. It would have been good had the authors explored Crumbs function better to improve the novelty aspect of their work. However, this reviewer understands that this would have been difficult to achieve given the ongoing COVID-19 crisis. I have not further comment on this manuscript.

Second revision

Author response to reviewers' comments

Reviewer 1 asked us to correct a typo error in the methods regarding gamma tubulin. We have done this.

They also asked us to consider adding a short discussion on the possible involvement of mechanical forces on apical junction formation. We have added a review on this at the start of the Discussion, and we have added a short paragraph that includes three additional primary papers on this at the end of the Discussion.

They also asked us to check the that Movies 2 to 4 open correctly. They are standard mp4 format and they open just fine for us.

The other two reviewers did not ask for any changes.

Third decision letter

MS ID#: DEVELOP/2020/191494

MS TITLE: Coordinated assembly and release of adhesions builds apical junctional belts during de novo polarisation of an epithelial tube

AUTHORS: Andrew C Symonds, Clare E Buckley, Charlotte A Williams, and Jonathan DW Clarke
ARTICLE TYPE: Research Article

© 2020. Published by The Company of Biologists under the terms of the Creative Commons Attribution License (https://creativecommons.org/licenses/by/4.0/).

I am happy to tell you that your manuscript has been accepted for publication in Development, pending our standard ethics checks.

© 2020. Published by The Company of Biologists under the terms of the Creative Commons Attribution License (https://creativecommons.org/licenses/by/4.0/).
